# Selective induction of human gut-associated acetogenic/butyrogenic microbiota based on specific microbial colonization of indigestible starch granules

**DOI:** 10.1038/s41396-022-01196-w

**Published:** 2022-02-03

**Authors:** Yusuke Nagara, Daichi Fujii, Toshihiko Takada, Mikiko Sato-Yamazaki, Toru Odani, Kenji Oishi

**Affiliations:** Microbiological Research Department, Yakult Central Institute, Kunitachi, Tokyo Japan

**Keywords:** Microbial ecology, Microbiota

## Abstract

Prediction of individualized responses is one of biggest challenges in dietary intervention to modulate human gut microbiota. Bacterial interspecies competition for dietary factors should underlie the inter-subject heterogeneity of microbial responses. Microscale localization of bacterial species around intestinal food structures could provide direct evidence for understanding this, however, little information is currently available. Here we analyzed human fecal sections and found multiple types of bacterial colonization of food structures. The most eminent one was dense and frequent colonization of starch granules by *Bifidobacterium adolescentis*. After intake of raw potato starch (pSt), *B. adolescentis* dramatically increased in every carrier of the species, accompanied by an increase in bifidobacterial metabolite acetate. In the other subjects, *Eubacterium rectale* and its metabolite butyrate increased, but it was suppressed in *B. adolescentis* carriers. A correlation analysis indicated the contribution of these species to respective metabolites. In vitro analyses of isolates of major gut bacterial species confirmed that these species are major colonizers of pSt and that *B. adolescentis* can colonize pSt even in the presence of the known starch granule–degrading bacterium *Ruminococcus bromii*. Collectively, we propose that specific binding of *B. adolescentis* or *E. rectale* to pSt selectively induces acetogenic or butyrogenic response of gut microbiota, where the former determines the response of the latter.

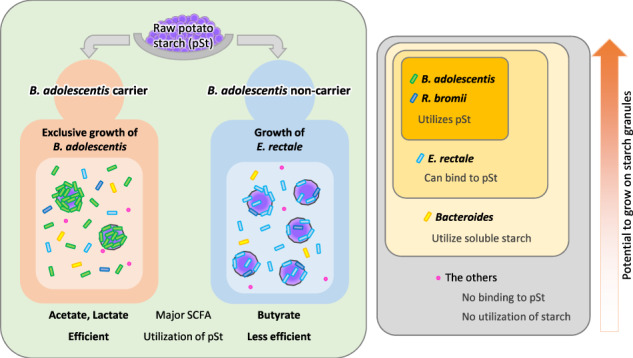

## Introduction

The intestinal microbiota contributes to many aspects of host health and diseases via bioactive metabolites including short-chain fatty acids (SCFAs). Diet is a rapid and powerful tool for modulating human microbiota and their metabolism [1–3]. A specific set of gut bacteria expands upon taking up each food, including indigestible polysaccharides, in some cases after degradation by host or microbial enzymes [4–7]. Direct utilization of each nutrient in the intestine by specific human gut–associated bacteria has been demonstrated using isolated strains and recently predicted from genomic data. However, it is still generally impossible to predict individualized responses to dietary intervention, and this will be the biggest limitation in the application of dietary intervention. To overcome this, it is necessary to thoroughly understand the nutritional ecology of gut bacteria, including specific competitions for each nutrient, and key behaviors that make bacteria more competitive in situ and enable them to take up nutrients before competitors consume them. A recent study showed a conceptual example of interspecies competition for a nutrient by using gnotobiotic mice [8], however, competitions and key behaviors are largely unidentified in vivo or in situ by analyzing normal human intestinal microbiota. To address this, we focused on the distribution of bacteria around food structures in human feces. We show specific bacterial colonization of a specific type of starch in human fecal sections, and also show the impact of the major colonizing species on the response of microbiota to a nutrient that is competed for by multiple gut bacterial species. We found that efficient utilization of starch granules was nearly monopolized by the major colonizing species *Bifidobacterium adolescentis*, although another colonizing species, *Eubacterium rectale*, also can bind and respond to the granules. The major gut fermentation product in each subject was determined by which of the two species responded and especially by the presence of *B. adolescentis*.

## Materials and methods

### Human studies and ethical approval

All human studies were conducted in accordance with the Declaration of Helsinki. The studies were scientifically and ethically reviewed and were approved by the Yakult Central Institute. Written informed consent was obtained from the participants after explanation of the study (aims, measurements, possible risks and consequences) before enrollment.

#### Experiment I

Five healthy male adults (age, 28–53 years) who had not taken antibiotics for at least one week prior to the day of the first sampling participated. Fecal specimens were collected twice with a 2-week interval. Subjects were asked to avoid overeating or overdrinking during the period. Contents of meals for 2 days before sampling were surveyed.

#### Experiment II

Ten healthy male adults (age, 29–56 years) were recruited. This experiment was designed according to a previous study [9]. After 10 days of pre-intake period, the participants were asked to consume raw potato starch (hereafter referred as pSt, unmodified potato starch, Bob’s Red Mill, Milwaukie, OR, USA) as follows: on the first day, one serving of 12 g; on the second day, one serving of 24 g; and then 48 g/day divided into equal 2 servings for 12 days. Fecal specimens were collected four times between the 3rd and 10th days of the pre-intake period and four times between the 4th and 14th days of the pSt intake period, with at least one-day interval between sampling in most cases. Intake of banana, any raw tubers, raw starch powder (other than that provided by the researcher), or large amounts of chilled potato salad was prohibited throughout the experiment to avoid introduction of large amounts of granular resistant starch to the intestine.

#### Experiment III

Ten healthy male adults (age, 28–54 years) were recruited. After 9 days of the pre-intake period, they were asked to take 40 g/day of raw Japanese yam (mix of *Dioscorea japonica* and *D. polystachya*, contains approx. 14% [w/w] of granular starch, Maruko Foods, Saitama, Japan) at lunch time for 4 days, followed by 8 days of the post-intake period (starchy foods are rarely consumed raw, but Japanese yam is an exceptionally common raw starchy food in Japan; after examining meal logs, it was the only candidate source of *B. adolescentis*-colonized starch granules in Experiment I). Six fecal specimens were collected in total from each subject (two for each period). Intake of banana and additional raw Japanese yam (other than that provided by the researcher) was prohibited during all periods of this experiment. Two subjects (F and G) were recruited again after the experiment, another informed consent was obtained, and fecal material was collected once for isolation of dominant bacteria. For this sampling, food intake was not restricted.

### Analysis of fecal sections

Fecal sections were prepared as described by Swidsinski et al. [10]. A fraction of fresh feces was sampled by subjects by puncturing it with a plastic straw (diameter 6 mm; length of the fecal specimen ~1–2 cm). The sample was kept cold using a cold pack (for up to 4 h) and fixed with methacarn fixative [11] for 2–4 h. The fixed sample was embedded first into 1% agarose and then into paraffin. Sections of 4 μm were obtained for the following analyses.

FISH staining was performed as previously described [12]. Briefly, sections were deparaffinized and incubated with a hybridization buffer containing 4.5 ng/μl of probes (Supplementary Table 1). To stain mucus (glycoproteins), sections were incubated with alcian blue at pH 2.5 and then with periodic acid–Schiff (PAS). Alcian blue was purchased from Merck Millipore (Burlington, MA, USA) and reagents for PAS staining from Muto Pure Chemicals (Tokyo, Japan). For iodine staining, sections were incubated with Lugol’s iodine solution (Muto Pure Chemicals) for 30 s and then washed with water for about 10 s.

Specimens were mounted using Vectashield with DAPI (Vector Laboratories, Burlingame, CA, USA) for fluorescent microscopy, or using Entellan New (Merck Millipore) for observation of iodine staining without FISH and of alcian blue–PAS staining. For double staining (FISH and iodine), specimens were first stained by FISH and then by iodine, and mounted using Vectashield with DAPI. Before mounting, more than ten independent fields of view containing one or more deep-purple starch granules were randomly selected for each slide and memorized by the microscope operating software MetaMorph (Molecular Devices, San Jose, CA, USA); immediately after mounting, observation of stained starch and image acquisition were performed using memorized positions (iodine staining fades soon after mounting, presumably because of redox reaction between mounting media and iodine; fading of starch granules is slower than that of soluble starch). Fluorescence was observed using a DM6000B microscope system (Leica Microsystems, Wetzlar, Germany). Monochrome images were acquired for each fluorescent dye and pseudocolors were assigned to make overlay images. Bright-field images for starch granule measurements were obtained under a BZX700 microscope (Keyence, Osaka, Japan).

To confirm the specificity of starch staining, serial sections were incubated with 0.5% porcine amylase (A3176, Sigma-Aldrich, St. Louis, MO, USA) in PBS at 37 °C for 2 h and then stained with Lugol’s solution.

A macro program that selects starch granules but not solubilized starch was designed in Image-Pro Plus 6 (Media Cybernetics, Rockville, MD, USA). Representative images containing average amounts of starch granules in each specimen were analyzed by this macro to calculate the percentage of starch granule area in the field. Area outside of fecal material and area occupied by air bubbles were omitted from the calculation. Area of excreted starch granules (%) was calculated as [area of starch granules during the intake period (%)] – [that during the pre-intake period (%)].

### Microbial profiling

Microbial profiling based on 16 S rRNA gene sequences was performed as previously described [13]. DNA was extracted from 20 mg of feces by bead–phenol method [14] and dissolved in 1 ml of TE buffer. The V1–V2 region of the 16 S rRNA gene was amplified by PCR using DNA (1 μl) as a template, TB Green Premix Ex Taq II (Tli RNaseH Plus) (Takara Bio, Shiga, Japan), and primers (27Fmod2-MiSeq and 338R-MiSeq, Supplementary Table 1). To prevent erroneous amplification, the reaction was stopped when the TB Green signal was close to saturation. The products were purified using Agencourt AMPure XP beads (Beckman Coulter, Brea, CA, USA) and all samples were pooled to make a library. The library was sequenced using a MiSeq Reagent Kit v2 (500 cycles) and a MiSeq instrument (Illumina, San Diego, CA, USA). Raw sequence data were deposited in DDBJ DRA under accession number PRJDB11235.

### Measurement of SCFAs

SCFAs were measured as previously described [6]. Fecal samples were suspended in a 9-fold volume of PBS, and an aliquot of the suspension was mixed with one-ninth volume of 10% perchloric acid. The mixture was tightly sealed and kept at 4 °C until analysis. On the day of analysis, the samples were filtered through Centricut Ultramini filters (#W-MO-045, Kurabo, Osaka, Japan) and analyzed by HPLC with pure SCFAs (lithium lactate, sodium acetate, sodium propionate and sodium butyrate) at known concentrations as standards.

### Measurement of total fecal bacterial count

Fecal samples were washed with PBS, resuspended in PBS containing 0.1% Tween-80, sonicated and centrifuged at 100 × *g* for 1 min to remove large debris. Aliquots of the supernatants were mounted using Vectashield with DAPI, and fluorescence of DAPI was imaged for ten independent fields of view using Leica DM6000B microscope. The cells in these images were counted using Image-Pro Plus 6.

### Isolation of major gut bacterial species

Fresh fecal samples or those stored at −80 °C were used. A small fraction of each sample was diluted and plated onto TOS propionate agar (Yakult Pharmaceutical Industry, Tokyo, Japan) supplemented with 50 μg/ml mupirocin, or BL agar (Nissui, Tokyo, Japan) supplemented with 5% defibrinated horse blood. Plates were incubated for 1–2 days at 37 °C in an anaerobic glove box. Colonies were sorted into types by their morphology. For each type, several single colonies were isolated and subjected to taxonomic identification by 16 S rRNA gene sequencing (Supplementary Table 1). Sequences were analyzed by NCBI BLAST against the bacterial 16 S rRNA gene database; 97% identity was used as a threshold to assign strains to species. Bifidobacterial isolates were additionally typed by RAPD (analysis of random amplified polymorphic DNA) using three primers (Supplementary Table 1). An isolate was chosen per RAPD type as a representative and was evaluated in vitro. For species other than bifidobacteria, a single strain was randomly chosen per species per subject and evaluated.

### In vitro evaluation of isolates

Binding to starch granules was analyzed as follows. Bacterial strains were grown anaerobically on modified GAM agar (Nissui, contains 0.5% soluble starch as the major carbon source). Approximately 1 μl of a colony was taken with an inoculating loop and suspended in 150 μl of PBS; then, 12 μl of this suspension, 80 μl of pSt suspension (1% w/v in PBS), and 88 μl of PBS were mixed by vortexing, and formation of bacteria–starch aggregates was examined. If aggregation occurred and the flocks fell immediately to the bottom of the tube making the supernatant transparent, the strain was judged positive. If no aggregation was observed, the strain was judged negative. In negative cases and in the negative control (no bacteria), visible precipitation of starch requires more than ~15 s. Strains that showed self-aggregation (i.e., could not be dispersed or aggregated without starch added) were categorized as undeterminable.

Starch-degrading ability was screened as follows. Strains were grown anaerobically on modified GAM agar, the culture plates were flooded with 1/2 diluted Lugol’s solution for about 30 s and then the solution was removed. Starch-degrading ability was judged from a clear zone around the colonies. When the diameter of this zone was <~1 mm or staining around the colony was reduced but was clearly visible, the strain was judged as weakly positive.

Utilization of pSt was analyzed as follows. The powder of GAM Semisolid without Dextrose (Nissui; does not contain starch) was thoroughly dissolved in water, agar was removed by precipitation, and the solution was autoclaved at 115 °C for 15 min. Broths containing different carbon sources were prepared by adding 1% w/v glucose, fructose, or pSt before autoclaving. All broths were introduced into an anaerobic chamber at least 1 or 2 days before use. A separate broth was prepared by adding 1% w/v pSt into the autoclaved broth without any additional carbon source, in an anaerobic chamber just before starting culture; starch used in this broth had been UV-irradiated in advance for at least 16 h. Bacterial strains were grown anaerobically on modified GAM agar. Each colony (~1 μl) was suspended in 150 μl PBS, and 5 μl was inoculated into each broth and incubated at 37 °C in the anaerobic chamber for 3 days, and then pH of the culture was measured.

### In vitro competition assay

*Bifidobacterium adolescentis* G202, *Ruminococcus bromii* YIT 6078 ^T^, and *R. bromii* ATCC 51896 were grown anaerobically on modified GAM broth at 37 °C. Fully grown cultures of either of two *R. bromii* strains and G202 were inoculated into GAM broth supplemented with 0.5% pSt (pSt-GAM) at an approximate 2:1 ratio and incubated for 24 h. Alternatively, *R. bromii* YIT 6078 ^T^ was grown on pSt-GAM broth for one day, then G202 was inoculated, and the resultant culture was incubated for 48 h.

Culture was sampled at the indicated time points. “Whole” samples were obtained by centrifuging the culture at 13,000 rpm for 10 s. “pSt-associated” samples were obtained as follows: the culture was centrifuged at 100 ×*g* for 10 s, the supernatant was removed, PBS was added to the pellet and resuspended, the sample was centrifuged as above and the supernatant was discarded. The pellets were heated at 95 °C for 15 min in TE, centrifuged at 13,000 rpm for 1 min, and the supernatants were used as PCR templates. PCR was performed using Premix Ex Taq Hot Start Version (Takara) and equal amounts of three primers: 27Fmod2-notag, g-Bifid-R, and Clept866mR (Supplementary Table 1). We confirmed that when the two species were mixed in arbitrary ratios after measuring OD, the corresponding PCR bands were detected in the expected ratio.

### Data analysis and statistics

Output fastq files were analyzed in QIIME2 software (version 2018.11) and its plugins [15]. Noise and chimeras were removed and the sequences were trimmed using the DADA2 plugin [16] with the following settings: denoise-paired --p-trim-left-f 20 --p-trim-left-r 17 --p-trunc-len-f 220 --p-trunc-len-r 200. For Experiment II, a total of 3.1 M reads were obtained (17K–76 K per sample, average 39 K), and for Experiment III, a total of 0.8 M reads were obtained (6K–36 K per sample, average 14 K). Amplicon sequence variants (ASVs) were classified in family level using the feature-classifier plugin [17] (classify-sklearn method). The classifier was trained on the SILVA138 database using the feature-classifier plugin [17] (fit-classifier-naive-bayes method). Most relevant species of ASVs were identified using vsearch [18] and the NCBI database 16 S RefSeq (BioProject IDs: 33175 and 33317, downloaded on 11 January 2019). ASVs were aligned using the R function AlignSeqs in the DECIPHER package [19]. A distance matrix was built from the aligned data using the DECIPHER function DistanceMatrix; this matrix was used to identify clusters using the DECIPHER function IdClusters with the “UPGMA” method and a cutoff value of 0.03 to make 97% OTUs. OTU representative ASVs were chosen by the highest abundance in the OTU and each OTU was labeled by vsearch result of the representative ASV. Because of high 16 S rRNA similarity (98.4%), OTUs classified as either *B. adolescentis* or *B. faecale* (or a retracted species *B. stercoris*) were denoted as *B. adolescentis* in this study.

OTUs whose abundance increased in response to pSt intake were identified using DESeq2 [20] with a design of “~ subject + period”, where “period” means the pre-intake or pSt intake period. Raw read counts were normalized by GMPR [21]. The log2 fold changes (LFC) were shrunk using DESeq2 function lfcShrink (type = “apeglm”) [22]. Since we used a threshold of 1 for the absolute LFC, the *s* value represents the “false sign or small” (FSOS) rate, where “small” denotes an absolute LFC less than 1. In identification of significantly increased OTUs, we analyzed the data from *B. adolescentis* carriers (subjects K, M, O, Q, R, T) and non-carriers (subjects L, N, P, S) separately.

To determine whether there was a significant change in SCFA concentration from the pre-intake period to the pSt intake period, we performed a Wilcoxon signed-rank test using the R-package exactRankTests [23] function wilcox.exact. We tested the data from *B. adolescentis* carriers and non-carriers separately and adjusted the *p* value using the Benjamini–Hochberg correction [24] for multiple comparisons.

Spearman’s rank correlation coefficient (Spearman’s ρ) and *p* value between the abundance of OTUs and SCFA concentrations were calculated using the R-package Hmisc [25] function rcorr. The GMPR-normalized OTU count data and SCFA concentration data from 80 samples were divided into 20 groups by subject and period (pre-intake or pSt intake), and averaged within each group. Spearman’s ρ values were also calculated between average OTU counts. A network plot was generated using the R-package visNetwork [26].

Excretion of starch granules (% area) was compared between *B. adolescentis* carriers and non-carriers using two-sided Welch’s *t* test.

## Results

**Fig. 1:**
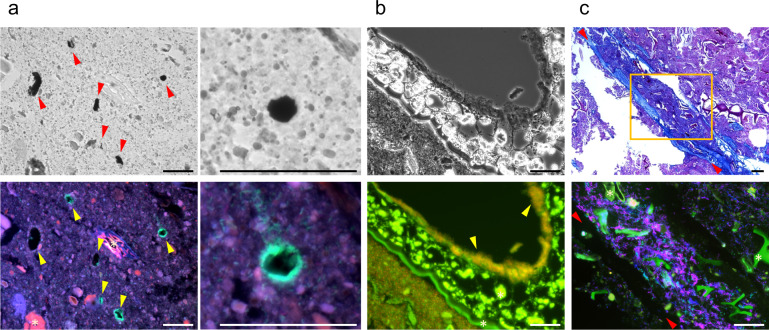
Microscopic survey of bacterial localization around materials in human feces. **a** Colonization of *B. adolescentis* on starch granules (arrowheads). Upper: iodine staining; Lower: FISH (Green: *B. adolescentis*, red: *B. catenulatum* group, blue: genus *Bifidobacterium*); *B. adolescentis* appears cyan because its green signal is merged with the blue signal from genus *Bifidobacterium*. Images on the right are high-power images of those on the left. **b** Colonization of *Lachnospiraceae* on plant tissue (arrowheads). Upper: phase contrast. Lower: FISH (Green: *Lachnospiraceae*, red: total bacteria). *Lachnospiraceae* appears yellow because its green signal is merged with the red signal from total bacteria. **c** Localization of *Bifidobacterium* and *Lachnospiraceae* near mucus layer (between arrowheads). Upper: Alcian blue PAS staining. Lower: Fluorescent image of a serial section corresponding to the rectangle in upper image (Green: genus *Bifidobacterium*, red: *Lachnospiraceae*, blue: total bacteria). Bars = 50 μm. *, Autofluorescence from plant-like structures.

To explore the nutrient-harvesting strategies of human gut bacteria in vivo, we examined the microscale localization of bacteria around intestinal nutrients (Experiment I). This method has been employed in a murine study and proven useful for elucidating how specific bacteria acquire nutrients in the intestine [12]. Stool samples were collected from five subjects who were taking habitual diet, and fecal paraffin sections were analyzed by fluorescence in situ hybridization (FISH) with probes specific for major bacterial taxa. Plant-like structures and bacteria were found in every specimen. Particle density differed substantially among subjects (Supplementary Fig. 1a and b). Several types of bacterial localization were found (Fig. 1 and Supplementary Fig. 1c). Food structures colonized by bacteria in some subjects were often not found in the others, reflecting diversity in diet. In every case, local accumulation of bacteria of the same taxa or morphology was evident, which was distinct from diffuse distribution (Supplementary Fig. 1d). The most eminent feature was colonization of bifidobacteria on undigested starch granules, which have been known as a type of resistant starch (resistant starch type 2: RS2) (Fig. 1a and Supplementary Fig. 1e). Granule surfaces were densely covered by bifidobacteria, which were identified as a starch-utilizing bacterium, *B. adolescentis* [27], by using a species-specific probe (Fig. 1a). We also found other features, for example, an unspecified clade of *Lachnospiraceae* localized along a plant exodermis–like cell layer (Fig. 1b), and some bifidobacteria and *Lachnospiraceae* bacteria enriched near the mucus layer (Fig. 1c).

**Fig. 2:**
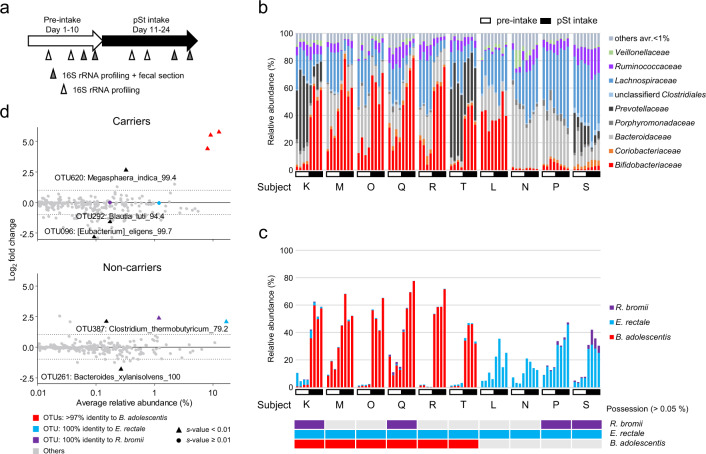
Two-week intake of potato starch results in expansion of *B. adolescentis*. **a** Scheme of the pSt intake experiment. **b** Family-level 16 S rRNA profiles before and during intake of pSt. Less frequent families (average relative abundance <1%) are shown as others. **c** Relative frequency of known major starch-utilizing species before and during intake of pSt. The presence (>0.05%; colored cells) or absence (gray cells) of the indicated species is shown beneath the graph. Note that *B. adolescentis* was responsible for a large fraction of the increase in *Bifidobacteriaceae*. **d** Response of OTUs to pSt. Only OTUs with >0.01% average relative abundance are plotted. Those that showed significant differences between the pre-intake and intake periods (*s* value < 0.01) are labeled by taxon name. The *s* value represents the “false sign or small” (FSOS) rate, where “small” denotes LogFC between −1 and 1, shown as dashed lines. Values following taxonomic name are % identity to the reference sequence. Three known starch-utilizing species are shown as colored dots.

**Fig. 3:**
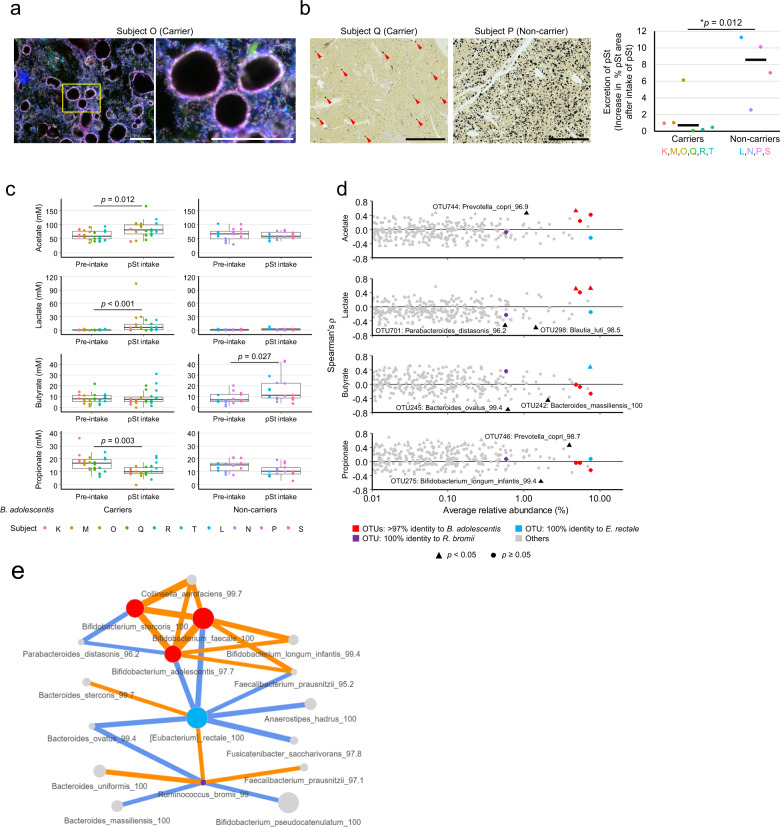
Metabolic responses of human gut microbiota to two-week intake of potato starch (pSt). **a** A representative image of colonizing *B. adolescentis* on fecal starch granules, which are visible as round dark areas (approx. diameter 20–50 μm). Green: *Bifidobacterium*, red: *B. adolescentis*, blue: total bacteria. Bar = 50 μm. **b** Excreted starch granules in fecal sections. Representative images of iodine-stained fecal sections during the pSt intake period from a *B. adolescentis* carrier and non-carrier. Difference in % area of excreted starch granules between the pre-intake and intake periods is shown in a graph. Horizontal bars indicate medians. * Two-tailed Welch’s *t* test. Arrowheads: starch granules (omitted in non-carrier). Bars = 1 mm. **c** SCFA concentrations before and during pSt intake in *B. adolescentis* carriers and non-carriers. Elements of box plots are as follows: horizontal bar, median; box limits, upper and lower quartiles; whiskers, 1.5× interquartile range. *P* values are based on Wilcoxon test and are adjusted using the Benjamini–Hochberg procedure. **d** Taxa that correlate highly with each SCFA as determined by Spearman’s correlation analysis. Given that the aim of this analysis was to reveal major producers of each SCFA, only OTUs with >0.5% average relative abundance and *p* < 0.05 are labeled. Three known starch-utilizing species are shown as colored dots. **e** Taxa that correlated (*p* < 0.05) with *B. adolescentis*, *E. rectale*, or *R. bromii* as determined by Spearman’s correlation analysis. Only OTUs with >0.5% average relative abundance and detected in more than half of the samples are shown. Orange: positive correlation, blue: negative correlation. Node size is proportional to the relative abundance of the OTU, and edge width is proportional to the correlation coefficient (Spearman’s ρ).

Bacterial colonization of a nutrient suggests that the nutrient is utilized by or serves as an important habitat for the bacteria. Dense colonization of starch granules by *B. adolescentis* led us to speculate that the species is a key degrader, which breaks down a large fraction of starch granules, possibly providing oligosaccharides, or a key consumer, which takes up and metabolizes a large fraction of starch granules, and affects the overall response of the microbiota to the nutrient. To test this hypothesis, we next checked the response of *B. adolescenti*s and whole-gut microbiota to intake of starch granules in ten healthy adults and evaluated how the presence or absence of the species affects the response of the microbiota (Experiment II; Fig. 2a). Commonly consumed purified potato starch (pSt) was used as a source of starch granules, as in a previous study [28]. Intake of 48 g/day pSt for 2 weeks did not considerably change total bacterial count (Supplementary Fig. 2), but it resulted in a massive increase in *Bifidobacteriaceae* including *B. adolescentis* in all *B. adolescentis* carriers (Subjects K, M, O, Q, R, T; Fig. 2b–d). Relative abundance of this species increased from 0.1%–28.9% to 32.0%–77.6% (Fig. 2c). Colonization of the species on starch granules was reproducible in every *B. adolescentis* carrier (Fig. 3a, Supplementary Fig. 3). In subjects without *B. adolescentis* (*B. adolescentis* non-carriers; Subjects L, N, P, S), the abundance of another known starch-utilizing species, *Eubacterium rectale* [29], increased substantially, from 2.7–15.2 to 12.6–45.9% (Fig. 2c). Yet another major known starch-utilizing species, *R. bromii* [30], also showed some increase in two subjects (Subjects P, S; Fig. 2c). A sporadic increase was also found in *Ruminococcus torques* (Supplementary Fig. 4).

Surprisingly, though all subjects had *E. rectale*, an increase in this species was observed in all four *B. adolescentis* non-carriers but not in any of the six carriers (Fig. 2c). Similarly, increases in *R. bromii* and *R. torques* were evident only in *B. adolescentis* non-carriers (Fig. 2c and Supplementary Fig. 4). However, since *B. adolescentis* and *E. rectale* were both highly abundant, their increase might lead to compositional problem: non-differential features might appear to be reduced due to the constant-sum constraint in relative abundance. To reduce these effects, we normalized the data by geometric mean of pairwise ratios (GMPR) method, and successfully confirmed that the same conclusion as above was obtained (Fig. 2d). Between *B. adolescentis* carriers and non-carriers, we found significant differences in gut microbiota metabolism. The amount of undigested pSt granules in fecal sections (Supplementary Fig. 5) was smaller in *B. adolescentis* carriers than in non-carriers (Fig. 3b). Furthermore, in *B. adolescentis* carriers but not in non-carriers, fecal concentrations of major bifidobacterial metabolites acetic and lactic acids increased and those of propionate decreased upon intake of pSt (Fig. 3c). Instead, a major metabolite of *E. rectale*, butyric acid, increased in *B. adolescentis* non-carriers but not in carriers (Fig. 3c). In Spearman’s correlation analysis, *B. adolescentis* was the most abundant among operational taxonomic units (OTUs) that were significantly correlated with the concentrations of acetic and lactic acids, and *E. rectale* was the most abundant among OTUs that were significantly correlated with the concentration of butyrate, suggesting that they produced a major fraction of each SCFA from pSt (Fig. 3d). In another Spearman’s correlation analysis, *E. rectale* was positively correlated with *R. bromii* and negatively with *B. adolescentis* and several butyrate-producing bacteria (Fig. 3e). For *B. adolescentis*, positive correlation was found with *B. longum* and *Collinsella aerofaciens*. Overall, these results strongly suggest that (i) among all bacterial species detected in this study, *B. adolescentis* most efficiently consumes intestinal pSt granules, which is facilitated by their colonization, to increase its abundance and generation of acetic and lactic acids, and that (ii) *E. rectale* can increase its abundance upon pSt intake, generating butyrate, but only in the absence of the dominant consumer *B. adolescentis*.

**Fig. 4:**
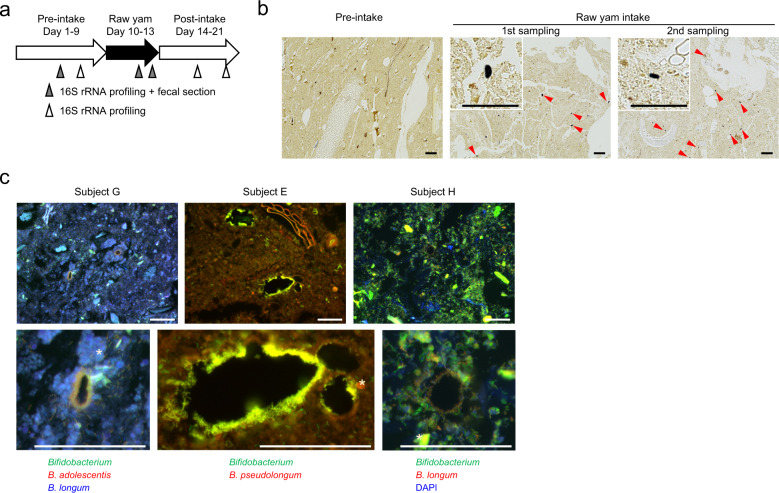
Colonization of bifidobacterial species on starch granules in feces. **a** Design of the raw yam intake experiment (ten subjects). **b** Starch granules were specifically detected during the raw yam intake period. Typical images of iodine-stained fecal sections are shown (specimens from subject B). Bars = 100 μm. Arrowheads: starch granules. **c** Colonization of bifidobacterial species on starch granules during the raw yam intake period. Bars = 50 μm. *, Autofluorescence.

To better understand the cause of the hierarchy between *B. adolescentis* and other species, especially *E. rectale*, we analyzed fecal sections again to distinguish colonizing and non-colonizing species (Experiment III). For this purpose, we employed another cohort, who consumed lower amounts of starch granules, to identify colonization more easily (Fig. 4a, b). This experiment confirmed that colonization of *B. adolescentis* on starch granules was outstanding among major bifidobacterial species (Table 1, Fig. 4c, Supplementary Fig. 6 and Supplementary note 1). Colonization by *B. adolescentis* was detected in every carrier of the species, and half or more of starch granules were densely colonized by the species. However, unfortunately we could not adequately determine the localization of *E. rectale* by rRNA-targeted FISH. In most of the specimens analyzed, the signal from *E. rectal* was too weak to identify its location. Presumably, the rRNA of this anaerobic species had already been degraded before fixation, when it was present in the rectum, or after the cells were damaged by exposure to oxygen at the moment of sampling.

**Table 1:** Determination of starch granule-colonizing bifidobacterial species.

Major species with average relative abundance >0.5% were analyzed. Colonization of the indicated taxa was examined using either or both of raw yam intake period samples. If no colonization was detected in a subject using *Bifidobacterium*-specific probe, species-level FISH for the subject was omitted. Bottom row: frequency of starch granules colonized by the indicated taxa among all granules; high: >65%, medium: 35–65%, low: <35%.

Therefore, instead, we checked the distribution of abilities to colonize and utilize pSt granules in major gut bacterial species by isolating them from two subjects who participated in Experiment III. A variety of dominant species including bifidobacteria were isolated, and pSt granule–binding ability and soluble starch–degrading ability were evaluated for at least one strain per species and subject (Supplementary Table 2). Strains with any positive results were included in further analysis of their ability to utilize pSt. *Bifidobacterium adolescentis* was able to bind to and utilize pSt granules (Table 2), as were most of other strains of the species from different origins (Supplementary Table 3 and Supplementary note 2). Among evaluated isolates other than *B. adolescentis*, the ability to utilize soluble (boiled) pSt was shared by many taxa; all evaluated *Bacteroidaceae* species and *E. rectale*. However, pSt granule–binding ability was shared only by *E. rectale* and *C. aerofaciens*, and it was strain-dependent (Table 2 and Supplementary Table 2). Surprisingly, none of the examined species other than *B. adolescentis*, including *E. rectale*, were able to degrade pSt granules. These results suggest that, among major gut bacterial species, both *B. adolescentis* and *E. rectale* are rare starch granule–binding species, and that the ability to efficiently utilize starch granules is nearly unique to *B. adolescentis*, whereas soluble starch can be utilized by many taxa including *Bacteroides*.

**Table 2:** Binding to and utilization of starch granules by major gut bacterial species.

Values indicates pH after 3-day incubation for culture with no carbon source (control), and the difference in pH between control and corresponding culture with 1% carbon source. As a positive control, either glucose (Glc) or fructose (Frc) was used. ++ strongly positive, + positive, – negative.

**Fig. 5:**
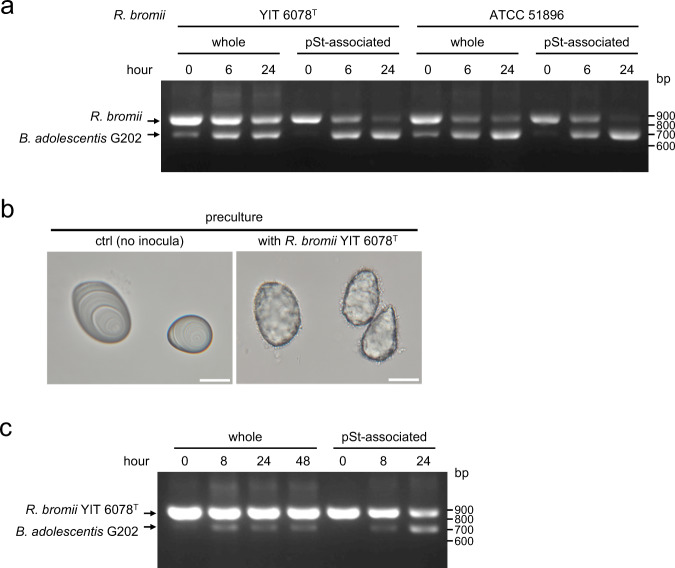
In vitro competition between *B. adolescentis* G202 and *R. bromii* strains. **a** *R. bromii* YIT 6078 ^T^ or *R. bromii* ATCC 51896 and *B. adolescentis* G202 were simultaneously inoculated into pSt-GAM broth and incubated for the indicated time. Relative abundance of two strains was determined by competitive PCR. **b**, **c** *R. bromii* YIT 6078^T^ was grown on pSt-GAM broth for one day and subsequently *B. adolescentis* G202 was inoculated and the cultures were incubated for the indicated time. **b** Bright-field images of pSt without bacteria and *R. bromii*-associated pSt just before inoculation of G202. Bars = 20 μm. **c** Relative abundance of the two strains was determined as in a. pSt was completely degraded and no pSt-associated fraction was obtained at 48 h.

Finally, since we were unable to obtain any isolates of the known RS2-degrading species *R. bromii* [30] in the above experiment, we compared the ability of commercially available *R. bromii* strains and the *B. adolescentis* strain obtained in this study to dominate the surface of pSt in vitro. When an *R. bromii* strain and a smaller amount of *B. adolescentis* G202 were inoculated together and incubated, the amounts of both strains in the whole culture became comparable, while G202 accounted for the majority of the population attached to pSt after 24 h of incubation (Fig. 5a). Even when *B. adolescentis* G202 was inoculated after *R. bromii* YIT 6078 ^T^ had densely covered the granular surface after one-day culture, the pSt-associated population after coculture contained a large amount of *B. adolescentis* G202, while *R. bromii* continued to constitute the majority of the whole culture (Fig. 5b, c). These results suggest that *B. adolescentis* is able to take over the surface of starch granules in the intestine, even in the presence of the known RS2-degrader *R. bromii*.

## Discussion

Bacterial strategies and processes for nutrient acquisition have not been surveyed by focusing on microscopic spatial information, at least for normal human gut bacteria. To uncover bacterial life in our gut, we applied histological methods to human fecal material and showed that a specific human-associated dominant bacterial species colonizes starch granules in vivo and effectively responds to their intake by the host. Our results suggest that activity of this species restrained other species with a potential to respond, and thereby determines the major SCFA generated in the gut.

We searched for the locations of major gut bacteria and found prominent and frequent colonization of *B. adolescentis* on starch granules in the human intestinal environment. Although adhesion of *B. adolescentis* onto starch granules has been demonstrated in vitro [31, 32] and ex vivo [33], we revealed selective, dense and frequent colonization in the normal human intestinal environment. This colonization was present in every subject who had taken pSt or raw yam starch granules and carried *B. adolescentis*, suggesting that the relationship between starch granules and this species is robust and important for adaptation of this species to human gut.

**Fig. 6:**
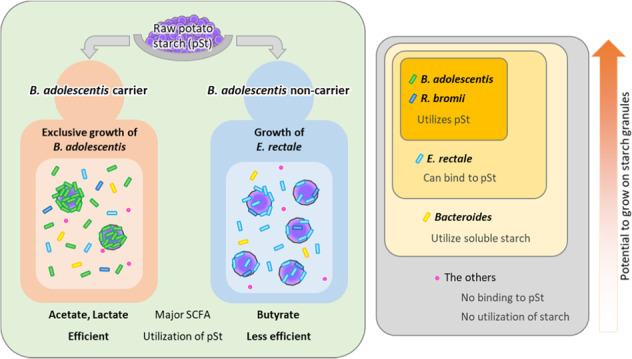
Proposed model for responses of human gut bacterial species to starch granules. Left: Two consequences of the gut microbiota after ingestion of pSt, which are determined by the posession of *B. adolescentis*. Right: Interspecies hierarchy of potential to grow efficiently using starch granules.

We found a remarkable increase in *B. adolescentis* abundance after pSt intake in each carrier of the species; the second largest response was found in the butyrate-producing bacterium *E. rectale*. Surprisingly, the response of *E. rectale* to pSt was observed in all *B. adolescentis* non-carriers but not in carriers, and a similar response was also observed in *R. bromii*. This relationship was consistently detected using GMPR method for normalization, which reduces the compositional problem. Given that the total bacterial count was comparable between the pre-intake and intake periods, the increase in the relative abundance of *B. adolescentis* should reflect an increase in its count. Despite the limited number of subjects, the difference between *B. adolescentis* carriers and non-carriers was evident (Fig. 6). The results also showed that the amount of pSt residues and the major SCFAs generated from pSt in the feces depended on whether the subject was a carrier or non-carrier of *B. adolescentis*. Consistently, correlation analysis suggested that *B. adolescentis*, the main increased species in the former, and *E. rectale*, that in the latter, contributed to production of acetic and lactic acids and of butyric acid, respectively. The decrease in propionate in carriers could be explained by a decrease in the abundance of the propionate-producing family *Bacteroidaceae* or *Prevotellaceae* (Fig. 2b) [34]. Though the responses of *B. adolescentis* and *E. rectale* to intake of starch granules or similar RS2 food have been reported [9, 28, 35], to our knowledge, this hierarchical relationship where *B. adolescentis* “rules” the activities of other less potent responding species has not been demonstrated. This inter-species relationship could help us predict individualized responses of gut microbiome to pSt and possibly to other starches categorized as RS2. Moreover, our results imply that a single species, *B. adolescentis*, can be a critical factor that determines the diversity of the health effects of starch granules among individuals. About half of the population is estimated to have *B. adolescentis* [36], and the effects of RS2 on metabolic health vary among trials and are not always significant [37, 38]. It is expected that ingestion or suppression (e.g., with a specific glycosidase inhibitor or antibody) of this species will unify the heterogeneous responses of the microbiota, SCFA, and possibly host metabolic parameters to starch granules. This approach will contribute to the efficient use of this potent and easy-to-use food material to fulfill individualized needs of a large fraction of the population. Considering that the levels of *B. adolescentis* and lactate in this study were quite high, the dose of starch granules has to be adjusted according to the desired individual effect. For example, using a low dose to maintain mild changes for a long time would be safe and suitable for metabolic effects, whereas using high doses to induce a large amount of acids may be effective for urgent eradication of some enteric pathogenic bacteria. In addition, potential differences between males and females in microbiota responses to starch granules remain to be addressed, since our data were obtained from male subjects only.

It should be considered that the relationship between *B. adolescentis* and *E. rectale* may be geographical region–specific or strain-specific, given that it was not evident in similar studies in the United States [9, 28, 35]. Indeed, a genomic study indicates that *E. rectale* strains can be divided into several geographically stratified types with different carbohydrate metabolism genes [39]. Future mechanistic and population studies will be necessary for understanding this. In addition to clarifying the relationship between *B. adolescentis* and *E. rectale*, our results imply downstream interspecies interactions. Positive correlation between *B. adolescentis* and *B. longum* suggests that the former species created an environment preferred by *Bifidobacterium* species, which led to the increase in their relative fitness. Positive correlation between *B. adolescentis* and *C. aerofaciens* suggests crossfeeding of maltose. Maltose is a substrate utilized by every strain of the latter species and can be enriched near *B. adolescentis*–colonized pSt. Our in vitro experiment suggests that some strains of *C. aerofaciens* bind to pSt, supporting this model. In *B. adolescentis* carriers, we detected no significant increases in butyrate, *E. rectale*, or other butyrate-producing bacteria. These bacteria can convert lactate to butyrate, but their activity seems to be suppressed in our experiment by the strongly acidic environment induced by metabolism of *B. adolescentis* [40]. Meanwhile, we detected an increase in a lactate-utilizing species *Megasphaera indica* [41] in *B. adolescentis* carriers (Fig. 2d, Correlation between OTUs of *B. adolescentis* and *M. indica*; *ρ* = 0.57, *p* < 0.01). This increase suggests that *M. indica* rather than butyrate producers could benefit from crossfeeding of lactate in a strongly acidic environment.

The advantage of *B. adolescentis* in utilization of starch granule is substantiated by in vitro activities of dominant species isolated from the participants. These results reinforce that *B. adolescentis* is highly adapted to raw granular starches and suggest that monopolization of the starch surface by adhesion promotes the selective response of the species as it limits expansion of other species, which can increase in abundance in the absence of *B. adolescentis*. The model is substantiated by the results of our in vitro competition assay, which suggest that *B. adolescentis* can take over the pSt surface from the known starch granule-degrading species, *R. bromii*. The acidic environment induced by *B. adolescentis* (especially the presence of lactate, pKa 3.86) may also contribute to the advantage of *B. adolescentis* over other species such as *E. rectale* [40]. We confirmed a previous result [30, 42] that *E. rectale* cannot utilize raw pSt in vitro, but it did respond to the intake of raw pSt. This may be explained by the activity of host amylase or amylases from other bacteria. Because *E. rectale* responded to pSt in the absence (or presence below the detection limit) of *R. bromii* (subjects L, N), there should be another mediator with activity similar to that of *R. bromii*.

Taken together, our results provide a good example of competition for a common but specific food ingredient and hierarchical relationship among major bacteria in the normal human intestine. Our findings could provide a basis for understanding similar interspecific relationship. In situ colonization analysis brought us mechanistic understanding of inter-species hierarchy in response to a nutrient and prompted us to propose future plans for a precise control of microbiota. A similar approach may reveal competition among other species for other nutrients and contribute to our understanding and ability to use the microbiota.

## Supplementary information

Supplementary notes

Supplementary figures

Supplementary tables
