# Supplementary material for: Selective induction of human gut-associated acetogenic/butyrogenic microbiota based on specific microbial colonization of indigestible starch granules
Source: ISME J. 2022 Feb 3;16(6):1502–11. doi: 10.1038/s41396-022-01196-w (PMC9123178; doi:10.1038/s41396-022-01196-w)
Supplement: Supplementary file 1 — Supplementary notes [file 41396_2022_1196_MOESM1_ESM.docx]

**Supplementary note 1**

By FISH staining of fecal sections, we confirmed that *Bifidobacterium* colonized the surface of pSt granules in six of the ten subjects participated in Experiment III, but not in the other four subjects **(Table 1)**. To further understand the localization at species level, we surveyed localization of major bifidobacterial species present in the six subject, and identified the colonizing species as *B. adolescentis* in four subjects, and as *B. pseudolongum* and *B. longum* in one subject each **(Table 1 and Fig. 4c)**. Colonization of *B. catenulatum* group was not detected. Colonization by *B. adolescentis* was detected in all four carriers of the species, while *B. longum* colonized in only one of the nine carriers (including four subjects without colonization of any *Bifidobacterium*) **(Table 1)**. Adhesion of *B. pseudolongum* was eminent in comparison with that of *B. longum* and was similar to that of *B. adolescentis* **(Fig. 4c)**; however, *B. pseudolongum* is not a common gut species in humans [43]. *B. adolescentis* and *B. pseudolongum* colonized many of the observed pSt granules, whereas *B. longum* in subject H colonized a relatively small fraction of pSt **(Table 1)**. The colonization of *B. pseudolongum* was consistent with the previous finding in murine intestine [12], suggesting evolutionally conserved relationship between bifidobacteria and RS2.

Major gut bacterial species were isolated from subjects F and G and subjected to analysis of pSt-binding and utilizing abilities **(Table 2** and **Supplementary Table 2)**.

**Supplementary note 2**

*In vitro* granular-starch-binding and -degrading abilities of *B. adolescentis* are reported to be strain dependent [31, 32]. However, due to a limited number of examined strains, it is difficult to discuss the strain-level distribution or detection rate of these abilities. Therefore, we evaluated 29 *B. adolescentis* strains isolated mainly from humans for the ability to bind to and utilize pSt. Of the 29 strains, 22 showed both binding and utilizing ability **(****Supplementary Table 3)**. Interestingly, the abilities to bind to and degrade pSt granules, and to degrade soluble (boiled) pSt were present as a set in most strains. The remaining seven strains, including the type strain, were unable to bind to or degrade granules, or their binding ability was undeterminable because of cell self-aggregation. Five of these seven strains were isolated in a single study performed in Germany [44] and therefore may be closely related to each other. The other two strains utilized soluble starch but were not active on raw pSt, suggesting that they lack key factors for utilizing starch granules. The result indicates that, though some strains are negative for the abilities to bind to and degrade starch granules, these abilities are broadly distributed and conserved among *B. adolescentis* strains, suggesting the importance of these abilities for their survival and acquiring nutrient.

**References**

43. Wong CB, Odamaki T, Xiao JZ. Insights into the reason of human-residential *Bifidobacteria* (HRB) being the natural inhabitants of the human gut and their potential health-promoting benefits. FEMS Microbiol Rev. 2020; 44: 369–385.

44. Reuter G. Vergleichende untersuchungen uber die bifidus-flora im sauglings- und erwachsenenstuhl. Zentralbl Bakteriol Orig. 1963; 191: 486–507.
