# Supplementary material for: Selective induction of human gut-associated acetogenic/butyrogenic microbiota based on specific microbial colonization of indigestible starch granules
Source: ISME J. 2022 Feb 3;16(6):1502–11. doi: 10.1038/s41396-022-01196-w (PMC9123178; doi:10.1038/s41396-022-01196-w)
Supplement: Supplementary file 2 — Supplementary figures [file 41396_2022_1196_MOESM2_ESM.pptx]

## Slide 1
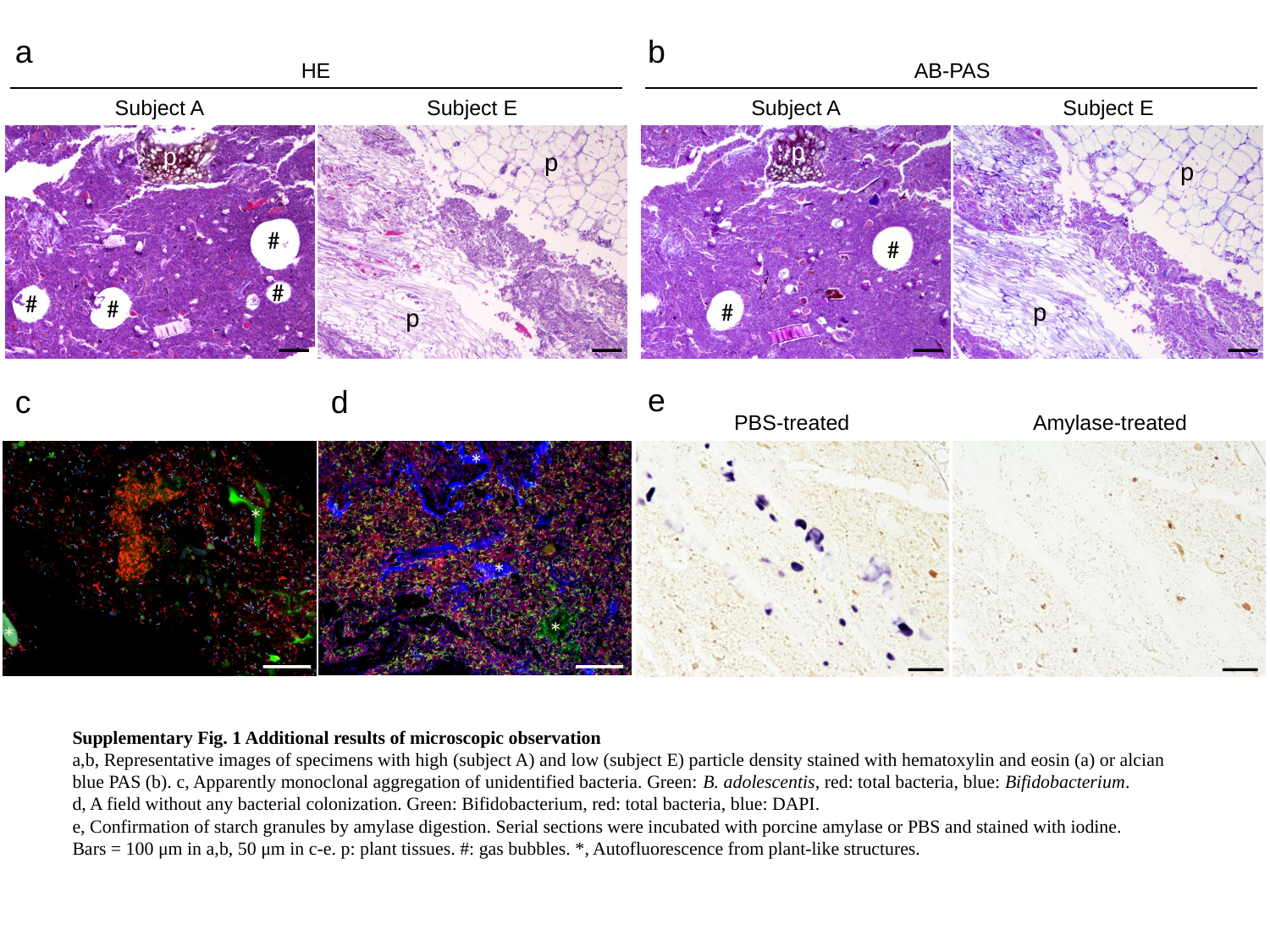

a
b
HE
AB-PAS
Subject A
p
#
#
#
#
Subject E
p
p
Subject A
p
#
#
Subject E
p
p
e
c
d
PBS-treated
Amylase-treated
*
*
*
*
*
Supplementary Fig. 1 Additional results of microscopic observation
a,b, Representative images of specimens with high (subject A) and low (subject E) particle density stained with hematoxylin and eosin (a) or alcian blue PAS (b). c, Apparently monoclonal aggregation of unidentified bacteria. Green: B. adolescentis, red: total bacteria, blue: Bifidobacterium.
d, A field without any bacterial colonization. Green: Bifidobacterium, red: total bacteria, blue: DAPI.
e, Confirmation of starch granules by amylase digestion. Serial sections were incubated with porcine amylase or PBS and stained with iodine.
Bars = 100 μm in a,b, 50 μm in c-e. p: plant tissues. #: gas bubbles. *, Autofluorescence from plant-like structures.

## Slide 2
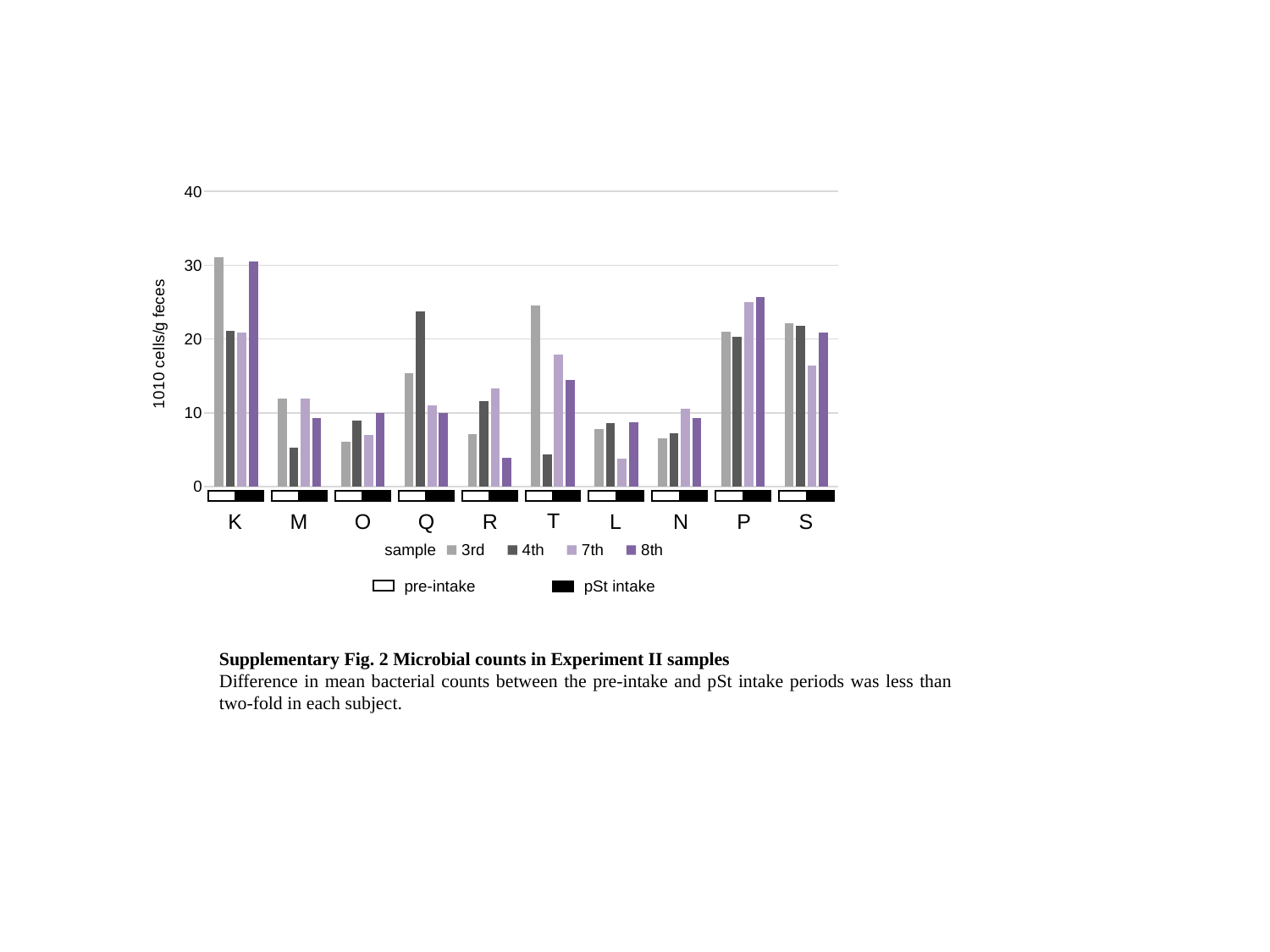

### Chart
| Category | 3rd | 4th | 7th | 8th |
|---|---|---|---|---|
| K | 31.058677 | 21.073351 | 20.8975702 | 30.4711134 |
| M | 11.970998 | 5.2620308 | 11.8985698 | 9.322892799999998 |
| O | 6.0603686 | 8.999814200000001 | 6.9856592 | 9.984512200000001 |
| Q | 15.3385024 | 23.7377322 | 11.0204796 | 9.9910226 |
| R | 7.0670392 | 11.5714222 | 13.3593408 | 3.9298402 |
| T | 24.5051456 | 4.398589 | 17.926386400000002 | 14.4319292 |
| L | 7.8596804 | 8.6482526 | 3.800446 | 8.7532328 |
| N | 6.5396968 | 7.2232888 | 10.5981174 | 9.2756924 |
| P | 20.9480258 | 20.2628062 | 25.0170258 | 25.6656244 |
| S | 22.0914148 | 21.8253022 | 16.4534084 | 20.896756400000005 |40
30
20
10
0
T
K
M
O
Q
R
L
N
P
S
sample
pre-intake
pSt intake
Supplementary Fig. 2 Microbial counts in Experiment II samples
Difference in mean bacterial counts between the pre-intake and pSt intake periods was less than two-fold in each subject.

## Slide 3
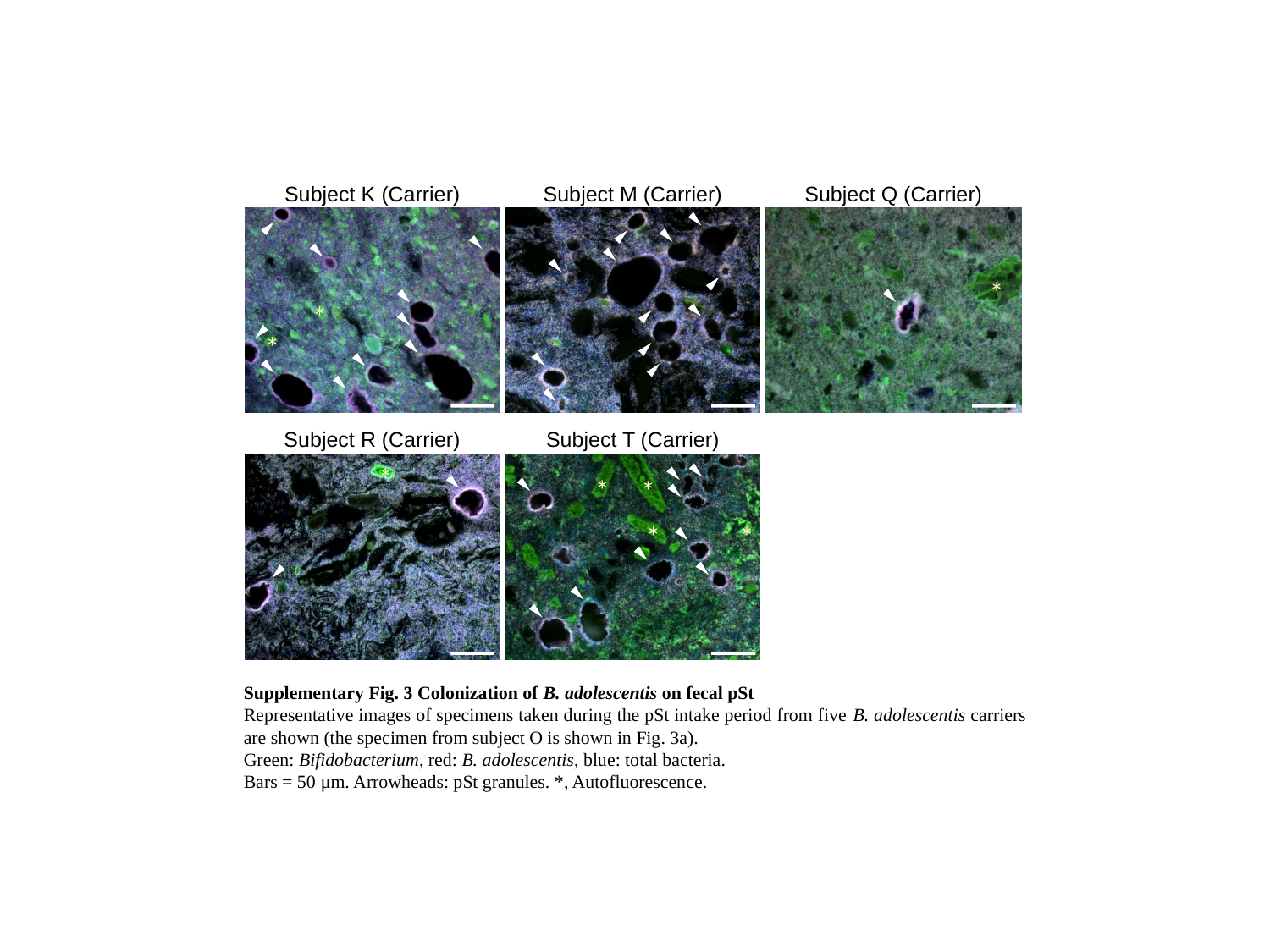

Subject K (Carrier)
Subject M (Carrier)
Subject Q (Carrier)
*
*
*
Subject R (Carrier)
Subject T (Carrier)
*
*
*
*
*
Supplementary Fig. 3 Colonization of B. adolescentis on fecal pSt
Representative images of specimens taken during the pSt intake period from five B. adolescentis carriers are shown (the specimen from subject O is shown in Fig. 3a).
Green: Bifidobacterium, red: B. adolescentis, blue: total bacteria.
Bars = 50 μm. Arrowheads: pSt granules. *, Autofluorescence.

## Slide 4
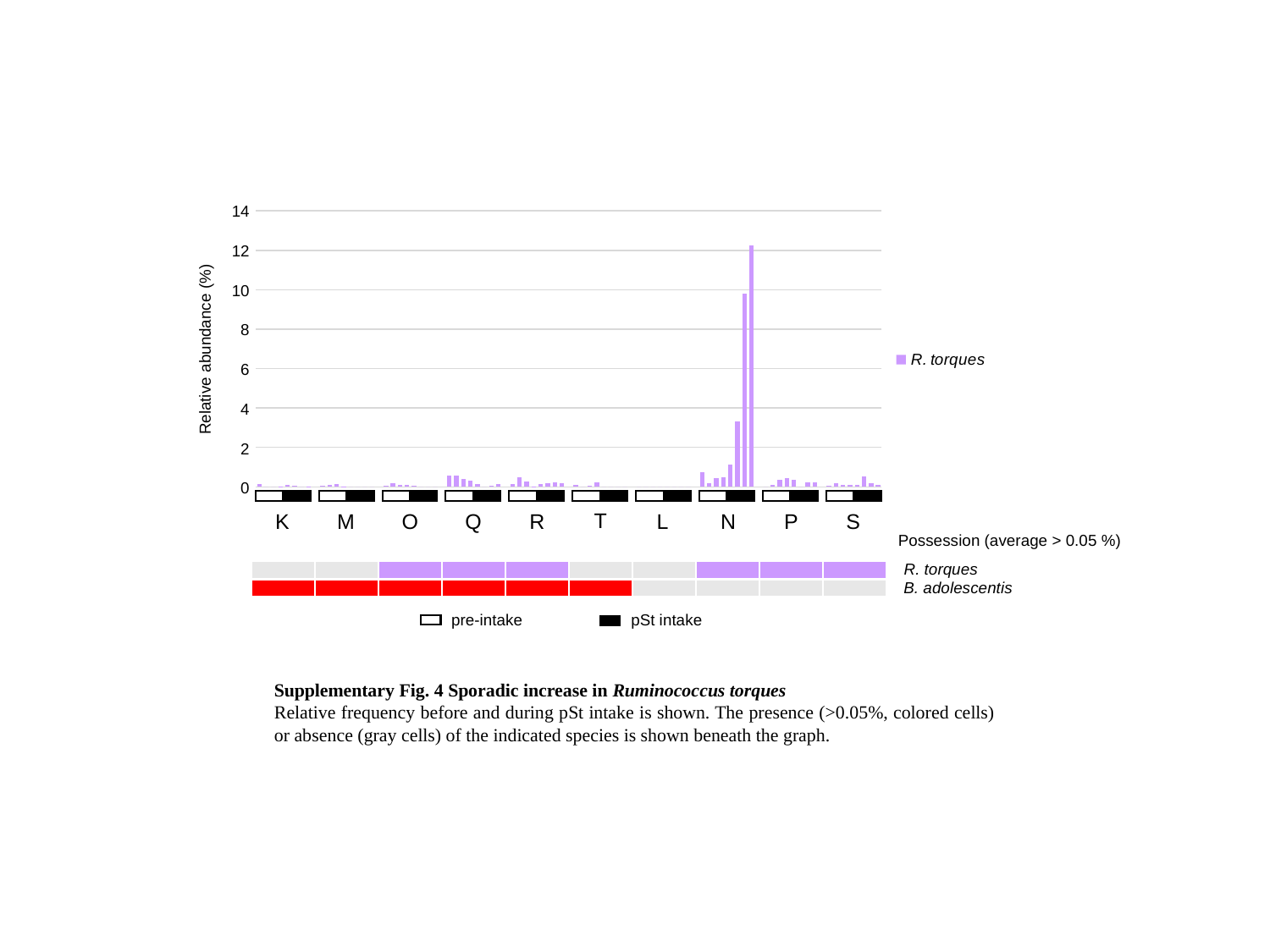

### Chart
| Category | R. torques |
|---|---|
| K1 | 0.00119723609495792 |
| K2 | 0.0 |
| K3 | 0.0 |
| K4 | 0.000143917432513003 |
| K5 | 0.00114743496130014 |
| K6 | 0.000331174565793347 |
| K7 | 0.0 |
| K8 | 0.000266560042649606 |
| | None |
| M1 | 0.000541201467257311 |
| M2 | 0.000879026429394643 |
| M3 | 0.00124010302394352 |
| M4 | 0.000210681554829874 |
| M5 | 0.0 |
| M6 | 0.0 |
| M7 | 0.0 |
| M8 | 0.0 |
| | None |
| O1 | 0.000553412711722287 |
| O2 | 0.00179109824173855 |
| O3 | 0.000784047805725668 |
| O4 | 0.00095746135959513 |
| O5 | 0.000362641347707117 |
| O6 | 0.0 |
| O7 | 0.0 |
| O8 | 0.0 |
| | None |
| Q1 | 0.00554620965198191 |
| Q2 | 0.00586838534599728 |
| Q3 | 0.00419551934826883 |
| Q4 | 0.00318618634051081 |
| Q5 | 0.00133022946458264 |
| Q6 | 0.0 |
| Q7 | 0.000616641615601032 |
| Q8 | 0.00142210246622506 |
| | None |
| R1 | 0.00136479954506681 |
| R2 | 0.00506437551966539 |
| R3 | 0.0025412960609911 |
| R4 | 0.00011314411327342 |
| R5 | 0.00152993714852795 |
| R6 | 0.00171722953634802 |
| R7 | 0.0024567610062893 |
| R8 | 0.00164511264451857 |
| | None |
| T1 | 0.00106396879024881 |
| T2 | 0.0 |
| T3 | 0.000587790746494248 |
| T4 | 0.00215640888677864 |
| T5 | 0.0 |
| T6 | 0.0 |
| T7 | 0.0 |
| T8 | 0.0 |
| | None |
| L1 | 0.0 |
| L2 | 0.0 |
| L3 | 0.0 |
| L4 | 0.0 |
| L5 | 0.0 |
| L6 | 0.0 |
| L7 | 0.0 |
| L8 | 0.0 |
| | None |
| N1 | 0.0074208693018325 |
| N2 | 0.00162905888827738 |
| N3 | 0.00424589593894694 |
| N4 | 0.00483376562131572 |
| N5 | 0.0110793733229464 |
| N6 | 0.0331352154531946 |
| N7 | 0.0978178539224526 |
| N8 | 0.122482508568676 |
| | None |
| P1 | 0.0 |
| P2 | 0.00102679946606427 |
| P3 | 0.00337622803376228 |
| P4 | 0.00428236566895059 |
| P5 | 0.00356330553449583 |
| P6 | 0.0 |
| P7 | 0.00231711353856338 |
| P8 | 0.00241965049492851 |
| | None |
| S1 | 0.00072130056039505 |
| S2 | 0.00174603174603174 |
| S3 | 0.000936969294735403 |
| S4 | 0.000793077316226361 |
| S5 | 0.000819869785387026 |
| S6 | 0.00509495136637332 |
| S7 | 0.00178675123955867 |
| S8 | 0.00106766806567999 |14
12
10
8
Relative abundance (%)
6
4
2
0
T
K
M
O
Q
R
L
N
P
S
Possession (average > 0.05 %)
R. torques
| | | | | | | | | | |
| --- | --- | --- | --- | --- | --- | --- | --- | --- | --- |
B. adolescentis
| | | | | | | | | | |
| --- | --- | --- | --- | --- | --- | --- | --- | --- | --- |
pre-intake
pSt intake
Supplementary Fig. 4 Sporadic increase in Ruminococcus torques
Relative frequency before and during pSt intake is shown. The presence (>0.05%, colored cells) or absence (gray cells) of the indicated species is shown beneath the graph.

## Slide 5
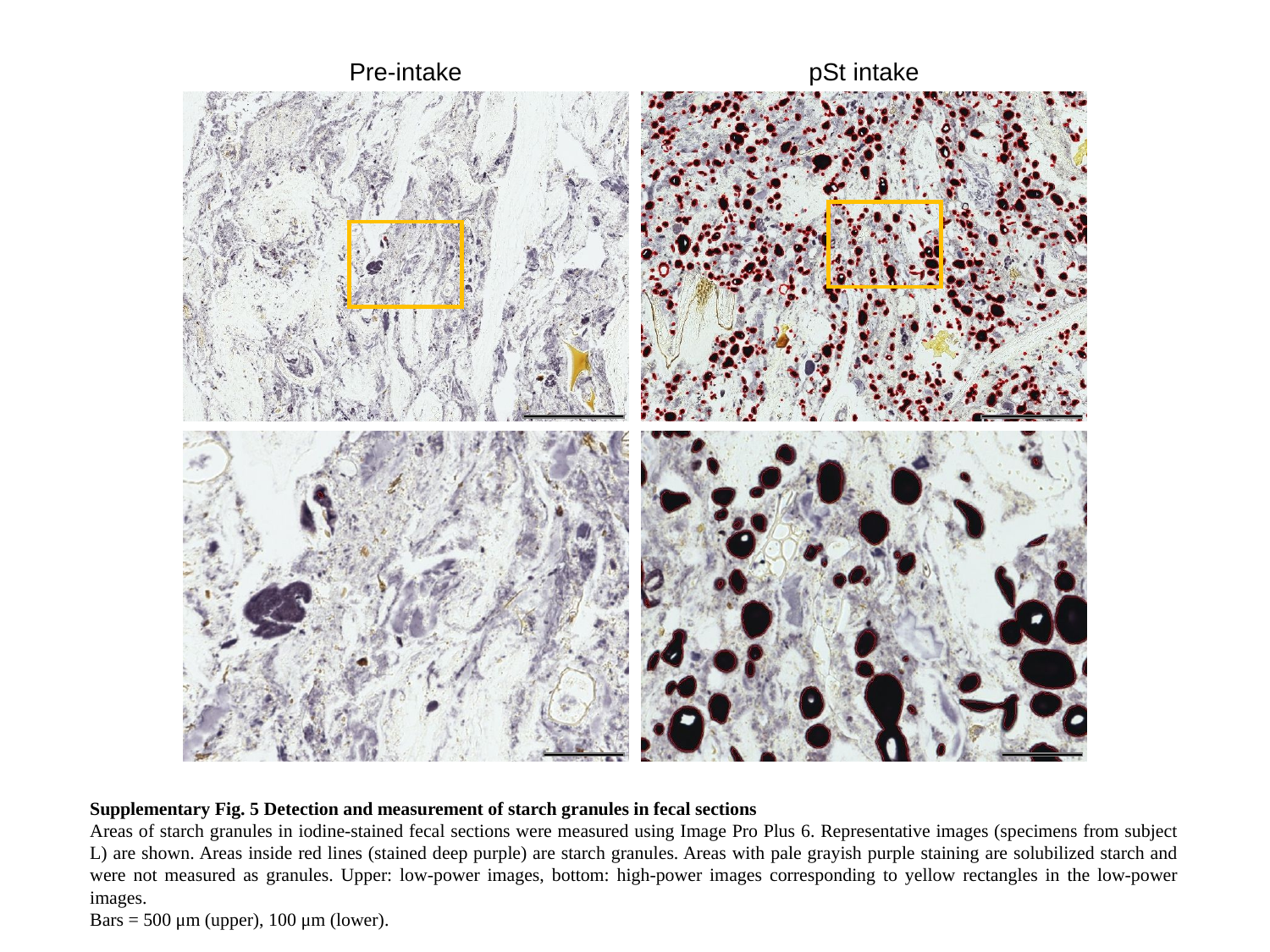

Pre-intake
pSt intake
Supplementary Fig. 5 Detection and measurement of starch granules in fecal sections
Areas of starch granules in iodine-stained fecal sections were measured using Image Pro Plus 6. Representative images (specimens from subject L) are shown. Areas inside red lines (stained deep purple) are starch granules. Areas with pale grayish purple staining are solubilized starch and were not measured as granules. Upper: low-power images, bottom: high-power images corresponding to yellow rectangles in the low-power images.
Bars = 500 μm (upper), 100 μm (lower).

## Slide 6
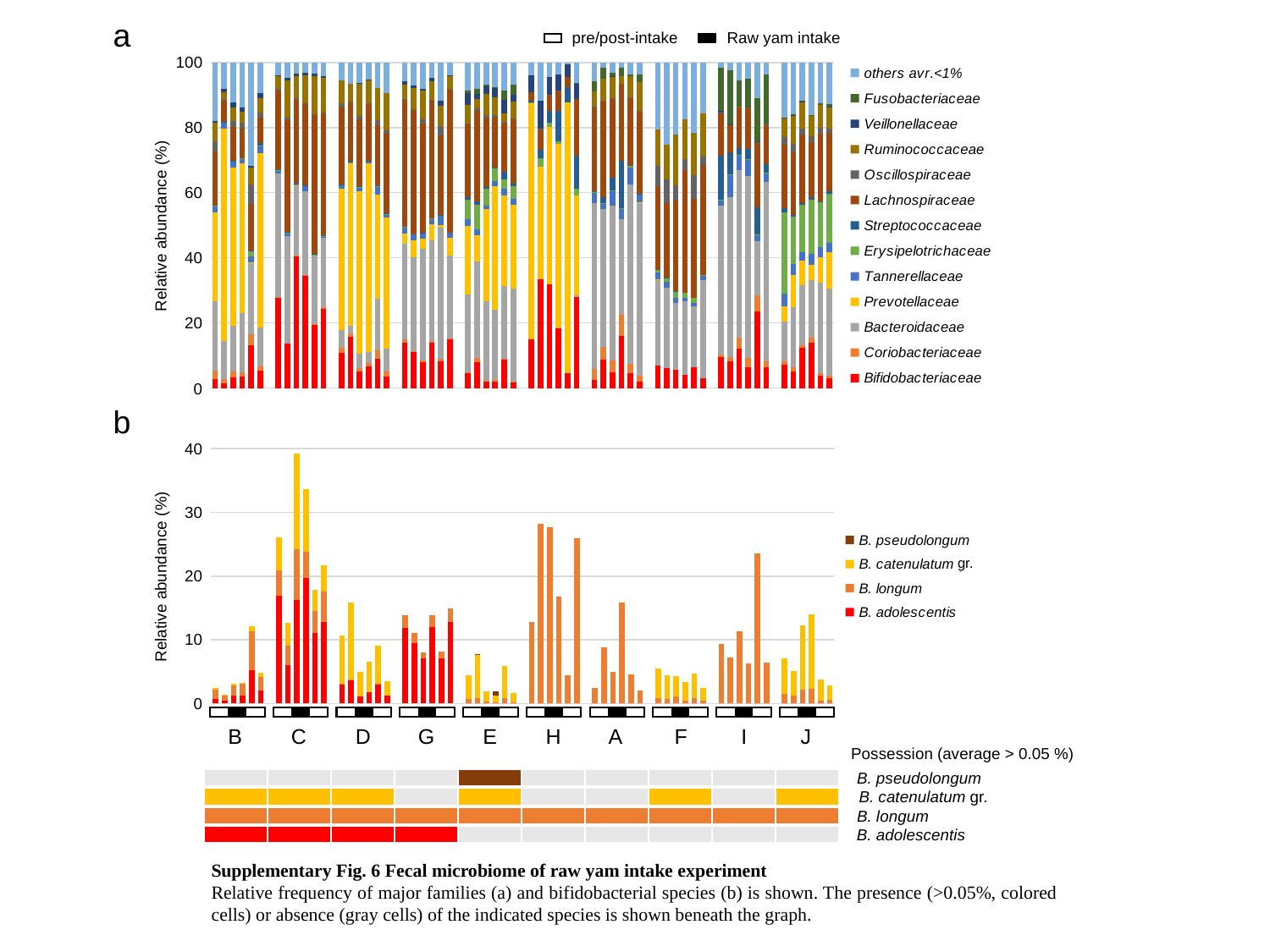

a
Raw yam intake
pre/post-intake
### Chart
| Category | Bifidobacteriaceae | Coriobacteriaceae | Bacteroidaceae | Prevotellaceae | Tannerellaceae | Erysipelotrichaceae | Streptococcaceae | Lachnospiraceae | Oscillospiraceae | Ruminococcaceae | Veillonellaceae | Fusobacteriaceae | others avr.<1% |
|---|---|---|---|---|---|---|---|---|---|---|---|---|---|
| B1 | 0.0265428727471 | 0.0277444019661 | 0.212561441835 | 0.273511742217 | 0.0162752594211 | 0.00480611687602 | 0.00218459858001 | 0.163626433643 | 0.0304751501912 | 0.0561441835063 | 0.00622610595303 | 0.0 | 0.17990169306405304 |
| B2 | 0.0146470621373 | 0.0120556588361 | 0.116218804574 | 0.654103994141 | 0.0190411807785 | 0.00185905019436 | 0.000676018252493 | 0.0650667568024 | 0.00276040786435 | 0.0214072446623 | 0.0108726268943 | 0.0 | 0.08129119486230502 |
| B3 | 0.0322626091857 | 0.0171879402649 | 0.140462102001 | 0.487461256692 | 0.0176105945337 | 0.00239504085658 | 0.000140884756269 | 0.104677373908 | 0.0187376725838 | 0.0410679064525 | 0.0156382079459 | 0.0 | 0.122358410819901 |
| B4 | 0.0360746720484 | 0.0123612512614 | 0.180373360242 | 0.461402623613 | 0.0137487386478 | 0.00138748738648 | 0.0 | 0.0915741675076 | 0.0184157416751 | 0.0325428859738 | 0.0150100908174 | 0.0 | 0.137108980827491 |
| B5 | 0.130151160698 | 0.0343260752204 | 0.220352708296 | 0.00179953212165 | 0.0165107072161 | 0.0169605902465 | 0.00143962569732 | 0.143107791974 | 0.0601943494691 | 0.0520064783156 | 0.00620838581969 | 0.0 | 0.3169425949251879 |
| B6 | 0.0530982683079 | 0.0129355309827 | 0.1190277488 | 0.535677029001 | 0.0220112664302 | 0.00208637596495 | 0.00792822866681 | 0.0789693302733 | 0.0135614437722 | 0.0453786772376 | 0.0151262257459 | 0.0 | 0.09419987481746699 |
| | None | None | None | None | None | None | None | None | None | None | None | None | None |
| C1 | 0.2767984233 | 0.00350377751013 | 0.377860505858 | 0.0 | 0.00930690901128 | 0.00218986094383 | 0.00109493047192 | 0.241979634293 | 0.00700755502026 | 0.0378845943283 | 0.00394174969889 | 0.0 | 0.038432059564226996 |
| C2 | 0.136993603412 | 0.0 | 0.330810234542 | 0.0 | 0.00778251599147 | 0.00202558635394 | 0.00149253731343 | 0.343710021322 | 0.00692963752665 | 0.113859275053 | 0.00980810234542 | 0.0 | 0.046588486140728004 |
| C3 | 0.404949381327 | 0.00149981252343 | 0.213473315836 | 0.00374953130859 | 0.00399950006249 | 0.0 | 0.00262467191601 | 0.254843144607 | 0.0042494688164 | 0.0682414698163 | 0.00749906261717 | 0.0 | 0.034870641169852 |
| C4 | 0.346658000217 | 0.000541653125339 | 0.257610226411 | 0.0 | 0.0161412631351 | 0.000649983750406 | 0.00140829812588 | 0.248835445781 | 0.00411656375257 | 0.0833062506771 | 0.00812479688008 | 0.0 | 0.032607518145395004 |
| C5 | 0.193968627749 | 0.00315241748642 | 0.209123020244 | 0.0 | 0.00319039841999 | 0.000455771202856 | 0.00269664628357 | 0.425272513198 | 0.00193702761214 | 0.119032245813 | 0.0067606061757 | 0.0 | 0.034410725815598994 |
| C6 | 0.244105713346 | 0.00285118982345 | 0.21500895566 | 0.0 | 0.00442299959791 | 0.000950396607815 | 0.00259531381365 | 0.372994114852 | 0.00241254523522 | 0.108601089301 | 0.00464232189202 | 0.0 | 0.041415359871291 |
| | None | None | None | None | None | None | None | None | None | None | None | None | None |
| D1 | 0.10725478816 | 0.0148578061521 | 0.0550203134068 | 0.434822983169 | 0.00998258850842 | 0.0 | 0.00243760882182 | 0.240046430644 | 0.00951828206616 | 0.0696459663378 | 0.0 | 0.0 | 0.05641323273356199 |
| D2 | 0.158150260954 | 0.00922442043937 | 0.0233037990047 | 0.498422138609 | 0.00291297487559 | 0.000182060929724 | 0.00467289719626 | 0.181636120888 | 0.00157786139095 | 0.0545575919408 | 0.0 | 0.0 | 0.065359873771118 |
| D3 | 0.0493244051178 | 0.0115389214397 | 0.0454980270238 | 0.49713021643 | 0.0132129618558 | 0.000657658734904 | 0.0038861652517 | 0.203455697716 | 0.00974530670812 | 0.101817529595 | 0.000358722946311 | 0.0 | 0.06337438718158699 |
| D4 | 0.065400115141 | 0.0115141047784 | 0.0338514680484 | 0.580656303972 | 0.00506620610248 | 0.0 | 0.000805987334485 | 0.173862982153 | 0.00460564191134 | 0.0715025906736 | 0.000345423143351 | 0.0 | 0.05238917674149699 |
| D5 | 0.0905358037056 | 0.0251377065598 | 0.158237356034 | 0.320881321983 | 0.0239359038558 | 0.000901352028042 | 0.0042063094642 | 0.183375062594 | 0.0150225338007 | 0.098948422634 | 0.0 | 0.0 | 0.078818227341048 |
| D6 | 0.0348861646235 | 0.0164623467601 | 0.0688616462347 | 0.402661996497 | 0.0108581436077 | 0.000350262697023 | 0.00252189141856 | 0.24707530648 | 0.00777583187391 | 0.113345008757 | 0.0 | 0.0 | 0.09520140105077 |
| | None | None | None | None | None | None | None | None | None | None | None | None | None |
| G1 | 0.138472435368 | 0.00979813481289 | 0.293589894936 | 0.0328178491323 | 0.0201865187109 | 0.000236099634046 | 0.0 | 0.390980993979 | 0.0025970959745 | 0.0439145319325 | 0.00944398536182 | 0.0 | 0.057962460158153 |
| G2 | 0.11035971223 | 0.00374100719424 | 0.288057553957 | 0.0507913669065 | 0.0195683453237 | 0.0 | 0.0 | 0.381294964029 | 0.00345323741007 | 0.0638848920863 | 0.00791366906475 | 0.0 | 0.07093525179845701 |
| G3 | 0.0801333140125 | 0.00463700912911 | 0.342921315751 | 0.0310099985509 | 0.0181857701782 | 0.000217359802927 | 0.000796985944066 | 0.334009563831 | 0.0124619620345 | 0.0896246920736 | 0.00412983625562 | 0.0 | 0.08187219243593602 |
| G4 | 0.139057396174 | 0.00408306112926 | 0.309729351377 | 0.0489967335511 | 0.0178488100793 | 0.000583294447037 | 0.0 | 0.361759216052 | 0.00349976668222 | 0.0578628091461 | 0.00828278114792 | 0.0 | 0.048296780214659996 |
| G5 | 0.0816020313943 | 0.00807940904894 | 0.403047091413 | 0.00877192982456 | 0.0285087719298 | 0.0 | 0.00161588180979 | 0.2452677747 | 0.0287396121884 | 0.060595567867 | 0.0154662973223 | 0.0 | 0.11830563250228102 |
| G6 | 0.149125894638 | 0.00293323946967 | 0.253314560601 | 0.0563181978177 | 0.0140795494544 | 0.0 | 0.0 | 0.442215182447 | 0.000938636630294 | 0.0382494426845 | 0.00363721694239 | 0.0 | 0.03918807931475401 |
| | None | None | None | None | None | None | None | None | None | None | None | None | None |
| E1 | 0.0442514907417 | 0.00439376503818 | 0.238518673501 | 0.210691494926 | 0.0207134637514 | 0.0596296683754 | 0.00857830316979 | 0.223245109321 | 0.00188304215922 | 0.0578512396694 | 0.0365100951982 | 0.00669526101057 | 0.08703839313734202 |
| E2 | 0.0785160123666 | 0.0133582220148 | 0.297730852243 | 0.0794493379222 | 0.0194831709736 | 0.0740243831301 | 0.0113165723619 | 0.277489354255 | 0.00705827451438 | 0.0299830834743 | 0.0149332088899 | 0.0166831943067 | 0.07997433354720798 |
| E3 | 0.0191452435188 | 0.00357956394403 | 0.244223885454 | 0.282026250136 | 0.0113895216401 | 0.0523375637271 | 0.0038507430307 | 0.214448421738 | 0.00688794880139 | 0.0660592255125 | 0.0234841089055 | 0.00498969519471 | 0.067577828397944 |
| E4 | 0.019536019536 | 0.004218004218 | 0.216894216894 | 0.380286380286 | 0.015873015873 | 0.036519036519 | 0.00510600510601 | 0.154068154068 | 0.004995004995 | 0.0545010545011 | 0.02886002886 | 0.00444000444 | 0.07470307470301302 |
| E5 | 0.0859427048634 | 0.00374750166556 | 0.223517654897 | 0.276898734177 | 0.0233177881412 | 0.027398401066 | 0.0244003997335 | 0.148317788141 | 0.00283144570286 | 0.0282311792139 | 0.0398067954697 | 0.0287308461026 | 0.08685876082610301 |
| E6 | 0.0168972969939 | 0.00266651696747 | 0.285429589918 | 0.257922362253 | 0.0171499143908 | 0.0389030791254 | 0.00934684368597 | 0.197013500996 | 0.0031717517613 | 0.0522356639627 | 0.0209391753445 | 0.0295000982401 | 0.06882420636034299 |
| | None | None | None | None | None | None | None | None | None | None | None | None | None |
| H1 | 0.149114801971 | 0.0 | 0.0 | 0.728235079394 | 0.0 | 0.000547545172477 | 0.00565796678226 | 0.0259171381639 | 0.0 | 0.0 | 0.0501003832816 | 0.0 | 0.040427085234521 |
| H2 | 0.33407191448 | 0.0 | 0.0 | 0.346588921283 | 0.0 | 0.0244897959184 | 0.0255782312925 | 0.0635957240039 | 0.00209912536443 | 0.0 | 0.0854810495627 | 0.0 | 0.11809523809527682 |
| H3 | 0.318709544847 | 0.0 | 0.0 | 0.482995993013 | 0.0 | 0.0124319326004 | 0.0359601356211 | 0.0500359601356 | 0.000513716223158 | 0.0 | 0.0547621493887 | 0.0 | 0.04459056817015 |
| H4 | 0.184662596096 | 0.0 | 0.000189819355247 | 0.563984940998 | 0.0 | 0.00819386883483 | 0.0935176690183 | 0.0612167420671 | 0.00072764086178 | 0.0 | 0.0510930431206 | 0.0 | 0.036413679648249 |
| H5 | 0.046080760095 | 0.0 | 0.0 | 0.831433095804 | 0.0 | 0.0 | 0.0452889944576 | 0.0323040380048 | 0.0 | 0.0 | 0.0391923990499 | 0.0 | 0.005700712589081 |
| H6 | 0.277979220417 | 0.0 | 0.00575273690817 | 0.307335611185 | 0.0 | 0.0206749877972 | 0.101771145666 | 0.173767519699 | 0.0 | 0.0 | 0.0497524579876 | 0.000139460288683 | 0.0628268600517 |
| | None | None | None | None | None | None | None | None | None | None | None | None | None |
| A1 | 0.0241803725084 | 0.033111861453 | 0.510619758196 | 0.0 | 0.0313691319028 | 0.00119812656573 | 0.00457466506916 | 0.258468576408 | 0.00163380895327 | 0.0450931271103 | 0.0 | 0.0327850996623 | 0.05696547217082801 |
| A2 | 0.0879637927944 | 0.038626994085 | 0.42229790285 | 0.0 | 0.0180139810002 | 0.000851407062198 | 0.0183724681843 | 0.294676465316 | 0.000627352572146 | 0.0696361355082 | 0.0 | 0.0315468721993 | 0.017386628428016002 |
| A3 | 0.0492357805061 | 0.0363317464295 | 0.473941368078 | 0.0 | 0.0446003507893 | 0.00350789275871 | 0.0383362565773 | 0.241919318467 | 0.00162866449511 | 0.0675269356051 | 0.000250563768479 | 0.0111500876973 | 0.031571034828362994 |
| A4 | 0.159196290572 | 0.0659637889159 | 0.294491057629 | 0.0 | 0.0306359019651 | 0.00171119452418 | 0.144954736145 | 0.237469640097 | 0.0 | 0.0236807242217 | 0.0 | 0.0261647162729 | 0.01573194965776 |
| A5 | 0.0463537155725 | 0.027264853629 | 0.552156591027 | 0.0 | 0.0508227957734 | 0.00498874068942 | 0.00436514810324 | 0.207309890871 | 0.000103932097696 | 0.0642646804088 | 0.0 | 0.00519660488481 | 0.037173046942659 |
| A6 | 0.02039408867 | 0.0168472906404 | 0.532413793103 | 0.000492610837438 | 0.0234482758621 | 0.00266009852217 | 0.00137931034483 | 0.251625615764 | 0.00108374384236 | 0.0891625615764 | 0.0 | 0.0243349753695 | 0.03615763546796599 |
| | None | None | None | None | None | None | None | None | None | None | None | None | None |
| F1 | 0.0675379090184 | 0.0 | 0.26705905826 | 0.000199521149242 | 0.0199521149242 | 0.00887869114126 | 0.00129688747007 | 0.254788507582 | 0.061851556265 | 0.113028731045 | 0.0 | 0.0 | 0.20540702314449097 |
| F2 | 0.0613016095171 | 0.0 | 0.247865640308 | 0.0 | 0.0176347095871 | 0.0110566829951 | 0.00111966410077 | 0.228411476557 | 0.0724982505248 | 0.106088173548 | 0.0 | 0.0 | 0.2540237928620371 |
| F3 | 0.0560220233999 | 0.0 | 0.205781142464 | 0.0 | 0.0159669649002 | 0.0195457673778 | 0.000963523743978 | 0.27832071576 | 0.0470750172058 | 0.155953200275 | 0.0 | 0.0 | 0.22037164487264096 |
| F4 | 0.0398476112026 | 0.0 | 0.226626853377 | 0.0 | 0.0108113673806 | 0.0140032948929 | 0.0 | 0.377471169687 | 0.0355230642504 | 0.120263591433 | 0.0 | 0.0 | 0.17545304777591203 |
| F5 | 0.0646274149034 | 0.0 | 0.186407543698 | 0.0 | 0.00977460901564 | 0.0162143514259 | 0.0 | 0.303932842686 | 0.0719871205152 | 0.131899724011 | 0.0 | 0.0 | 0.215156393744358 |
| F6 | 0.0308862510084 | 0.0 | 0.299527486458 | 0.0 | 0.0127924397833 | 0.00276593292613 | 0.0 | 0.338826783451 | 0.0260458683877 | 0.132188544428 | 0.0 | 0.0 | 0.15696669355767204 |
| | None | None | None | None | None | None | None | None | None | None | None | None | None |
| I1 | 0.0940809274328 | 0.00958644521235 | 0.45758555345 | 0.0 | 0.0150484895775 | 0.00211793557017 | 0.135547876491 | 0.130197302419 | 0.0016720543975 | 0.0 | 0.00613086612418 | 0.132872589455 | 0.0151599598707 |
| I2 | 0.0828014964789 | 0.0119938380282 | 0.490867077465 | 0.0 | 0.0684969190141 | 0.00181558098592 | 0.0684969190141 | 0.0829665492958 | 0.00335607394366 | 0.0 | 0.000110035211268 | 0.16494278169 | 0.024152728873243996 |
| I3 | 0.120712452361 | 0.0341448238314 | 0.514583495372 | 0.0 | 0.0458893987711 | 0.00178890876565 | 0.020611340126 | 0.12382359804 | 0.00381115345726 | 0.00015555728397 | 0.0 | 0.0797231080345 | 0.054756163957297994 |
| I4 | 0.0637969094923 | 0.0289183222958 | 0.559768211921 | 0.0 | 0.0480684326711 | 0.0016004415011 | 0.0333333333333 | 0.123620309051 | 0.00358719646799 | 0.000165562913907 | 0.000496688741722 | 0.087472406181 | 0.049172185430474 |
| I5 | 0.236302142052 | 0.0479143179256 | 0.166629086809 | 0.0 | 0.0188275084555 | 0.00259301014656 | 0.0836527621195 | 0.197294250282 | 0.000789177001127 | 0.0 | 0.00372040586246 | 0.133032694476 | 0.109244644870389 |
| I6 | 0.0645595158036 | 0.0163468004759 | 0.551445864156 | 0.0 | 0.0284517096891 | 0.00206921525012 | 0.0247788526201 | 0.120531788319 | 0.00222440639388 | 0.0 | 0.0 | 0.153897884227 | 0.035693963064549 |
| | None | None | None | None | None | None | None | None | None | None | None | None | None |
| J1 | 0.0716286514606 | 0.0106042416967 | 0.121982126184 | 0.047552354275 | 0.0372815792984 | 0.249966653328 | 0.0141389889289 | 0.193677470988 | 0.0238095238095 | 0.0579565159397 | 0.0 | 0.00206749366413 | 0.169334400426881 |
| J2 | 0.05150942166 | 0.01212753472 | 0.185042707179 | 0.098780726348 | 0.0337745321771 | 0.146378040034 | 0.00410771337289 | 0.191758492534 | 0.0252330964335 | 0.0886744474147 | 0.000326008997848 | 0.00202125578666 | 0.16026602334234 |
| J3 | 0.123258513932 | 0.00909442724458 | 0.183533281734 | 0.0756578947368 | 0.0266060371517 | 0.144349845201 | 0.00570820433437 | 0.210042569659 | 0.0183823529412 | 0.0832043343653 | 0.000580495356037 | 0.00135448916409 | 0.11822755417962101 |
| J4 | 0.140091222191 | 0.0148003351019 | 0.176207763195 | 0.0467281020199 | 0.0345341152378 | 0.164944615098 | 0.00800521269664 | 0.169598808526 | 0.0174997672903 | 0.0656241273387 | 0.0 | 0.000930838685656 | 0.161035092618363 |
| J5 | 0.0372771474878 | 0.0080226904376 | 0.277147487844 | 0.0784440842788 | 0.0321717990276 | 0.136466774716 | 0.00380875202593 | 0.2085089141 | 0.0175040518639 | 0.0698541329011 | 0.0 | 0.00526742301459 | 0.12552674230142902 |
| J6 | 0.0286744505495 | 0.00858516483516 | 0.26768543956 | 0.112809065934 | 0.0286744505495 | 0.151270604396 | 0.00549450549451 | 0.180975274725 | 0.0130494505495 | 0.0666208791209 | 0.00051510989011 | 0.00772664835165 | 0.12791895604401 |100
80
60
Relative abundance (%)
40
20
### Chart
| Category | B. adolescentis | B. longum | B. catenulatum gr. | B. pseudolongum |
|---|---|---|---|---|
| B1 | 0.00775532495903878 | 0.0146368104860732 | 0.00229382850901147 | 0.0 |
| B2 | 0.00467579291307532 | 0.007717875049293 | 0.00135203650498563 | 0.0 |
| B3 | 0.0122569737954353 | 0.0164130741053818 | 0.00183150183150183 | 0.0 |
| B4 | 0.0124873864783047 | 0.0182896064581231 | 0.0018920282542886 | 0.0 |
| B5 | 0.0523663847399676 | 0.0616789634694979 | 0.00742307000179953 | 0.0 |
| B6 | 0.0199248904652618 | 0.0215939912372209 | 0.0063634466930941 | 0.0 |
| | None | None | None | None |
| C1 | 0.169495237052447 | 0.0391985108945582 | 0.0515712252271981 | 0.0 |
| C2 | 0.0607675906183369 | 0.0300639658848614 | 0.0360341151385928 | 0.0 |
| C3 | 0.162104736907887 | 0.0801149856267967 | 0.149481314835646 | 0.0 |
| C4 | 0.19651175387282 | 0.0422489437764056 | 0.0980392156862745 | 0.0 |
| C5 | 0.110904326028334 | 0.0346006304834973 | 0.0324736982035018 | 0.0 |
| C6 | 0.127499360309975 | 0.0481777972730928 | 0.0409767152831085 | 0.0 |
| | None | None | None | None |
| D1 | 0.0302959953569356 | 0.00127684271619269 | 0.0756819500870575 | 0.0 |
| D2 | 0.0362301250151717 | 0.00151717441437068 | 0.120402961524457 | 0.0 |
| D3 | 0.0112399856510821 | 0.0 | 0.0380844194666986 | 0.0 |
| D4 | 0.0177317213586644 | 0.0 | 0.0476683937823834 | 0.0 |
| D5 | 0.0294441662493741 | 0.00170255383074612 | 0.0593890836254382 | 0.0 |
| D6 | 0.0123292469352014 | 0.0 | 0.0225569176882662 | 0.0 |
| | None | None | None | None |
| G1 | 0.118167866839806 | 0.0203045685279188 | 0.0 | 0.0 |
| G2 | 0.0943884892086331 | 0.0159712230215827 | 0.0 | 0.0 |
| G3 | 0.070569482683669 | 0.00956383132879293 | 0.0 | 0.0 |
| G4 | 0.120158656089594 | 0.0188987400839944 | 0.0 | 0.0 |
| G5 | 0.0714450600184672 | 0.0101569713758079 | 0.0 | 0.0 |
| G6 | 0.128710547929133 | 0.0204153467089053 | 0.0 | 0.0 |
| | None | None | None | None |
| E1 | 0.0 | 0.00711371482372633 | 0.0371377759179831 | 0.0 |
| E2 | 0.0 | 0.0084582628478096 | 0.069416088199265 | 0.000291664236131366 |
| E3 | 0.0 | 0.00265755504935459 | 0.0164876884694652 | 0.0 |
| E4 | 0.0 | 0.00210900210900211 | 0.0101010101010101 | 0.00688200688200688 |
| E5 | 0.0 | 0.00816122584943371 | 0.0507161892071952 | 0.0 |
| E6 | 0.0 | 0.00247003676986555 | 0.0139781626294664 | 0.0 |
| | None | None | None | None |
| H1 | 0.0 | 0.127395510129586 | 0.0 | 0.0 |
| H2 | 0.0 | 0.282565597667639 | 0.0 | 0.0 |
| H3 | 0.0 | 0.276276584814548 | 0.0 | 0.0 |
| H4 | 0.0 | 0.167673763801449 | 0.0 | 0.0 |
| H5 | 0.0 | 0.0446555819477435 | 0.0 | 0.0 |
| H6 | 0.0 | 0.260023708249076 | 0.0 | 0.0 |
| | None | None | None | None |
| A1 | 0.0 | 0.0241803725084413 | 0.0 | 0.0 |
| A2 | 0.0 | 0.0879637927944076 | 0.0 | 0.0 |
| A3 | 0.0 | 0.0492357805061388 | 0.0 | 0.0 |
| A4 | 0.0 | 0.15919629057187 | 0.0 | 0.0 |
| A5 | 0.0 | 0.0463537155724926 | 0.0 | 0.0 |
| A6 | 0.0 | 0.0203940886699507 | 0.0 | 0.0 |
| | None | None | None | None |
| F1 | 0.0 | 0.0091779728651237 | 0.0458898643256185 | 0.0 |
| F2 | 0.0 | 0.00769769069279216 | 0.0361091672498251 | 0.0 |
| F3 | 0.0 | 0.0105987611837577 | 0.0330350997935306 | 0.0 |
| F4 | 0.0 | 0.00483937397034596 | 0.0287273476112026 | 0.0 |
| F5 | 0.0 | 0.00793468261269549 | 0.0394434222631095 | 0.0 |
| F6 | 0.0 | 0.00426414659444509 | 0.0205140025354385 | 0.0 |
| | None | None | None | None |
| I1 | 0.0 | 0.0940809274328391 | 0.0 | 0.0 |
| I2 | 0.0 | 0.0717429577464789 | 0.0 | 0.0 |
| I3 | 0.0 | 0.114101267791864 | 0.0 | 0.0 |
| I4 | 0.0 | 0.0626379690949227 | 0.0 | 0.0 |
| I5 | 0.0 | 0.235400225479143 | 0.0 | 0.0 |
| I6 | 0.0 | 0.0645595158036315 | 0.0 | 0.0 |
| | None | None | None | None |
| J1 | 0.0 | 0.014672535680939 | 0.0569561157796452 | 0.0 |
| J2 | 0.0 | 0.0123883419182369 | 0.0391210797418009 | 0.0 |
| J3 | 0.0 | 0.0217685758513932 | 0.101489938080495 | 0.0 |
| J4 | 0.0 | 0.0225262961928698 | 0.117564925998324 | 0.0 |
| J5 | 0.0 | 0.00470016207455429 | 0.0325769854132901 | 0.0 |
| J6 | 0.0 | 0.00549450549450549 | 0.0231799450549451 | 0.0 |0
b
40
30
20
Relative abundance (%)
10
0
H
B
C
D
G
E
A
F
I
J
gr.
Possession (average > 0.05 %)
B. pseudolongum
| | | | | | | | | | |
| --- | --- | --- | --- | --- | --- | --- | --- | --- | --- |
B. catenulatum gr.
| | | | | | | | | | |
| --- | --- | --- | --- | --- | --- | --- | --- | --- | --- |
B. longum
| | | | | | | | | | |
| --- | --- | --- | --- | --- | --- | --- | --- | --- | --- |
B. adolescentis
| | | | | | | | | | |
| --- | --- | --- | --- | --- | --- | --- | --- | --- | --- |
Supplementary Fig. 6 Fecal microbiome of raw yam intake experiment
Relative frequency of major families (a) and bifidobacterial species (b) is shown. The presence (>0.05%, colored cells) or absence (gray cells) of the indicated species is shown beneath the graph.
