# Supplementary material for: Selective induction of human gut-associated acetogenic/butyrogenic microbiota based on specific microbial colonization of indigestible starch granules
Source: ISME J. 2022 Feb 3;16(6):1502–11. doi: 10.1038/s41396-022-01196-w (PMC9123178; doi:10.1038/s41396-022-01196-w)
Supplement: Supplementary file 3 — Supplementary tables [file 41396_2022_1196_MOESM3_ESM.pptx]

## Slide 1
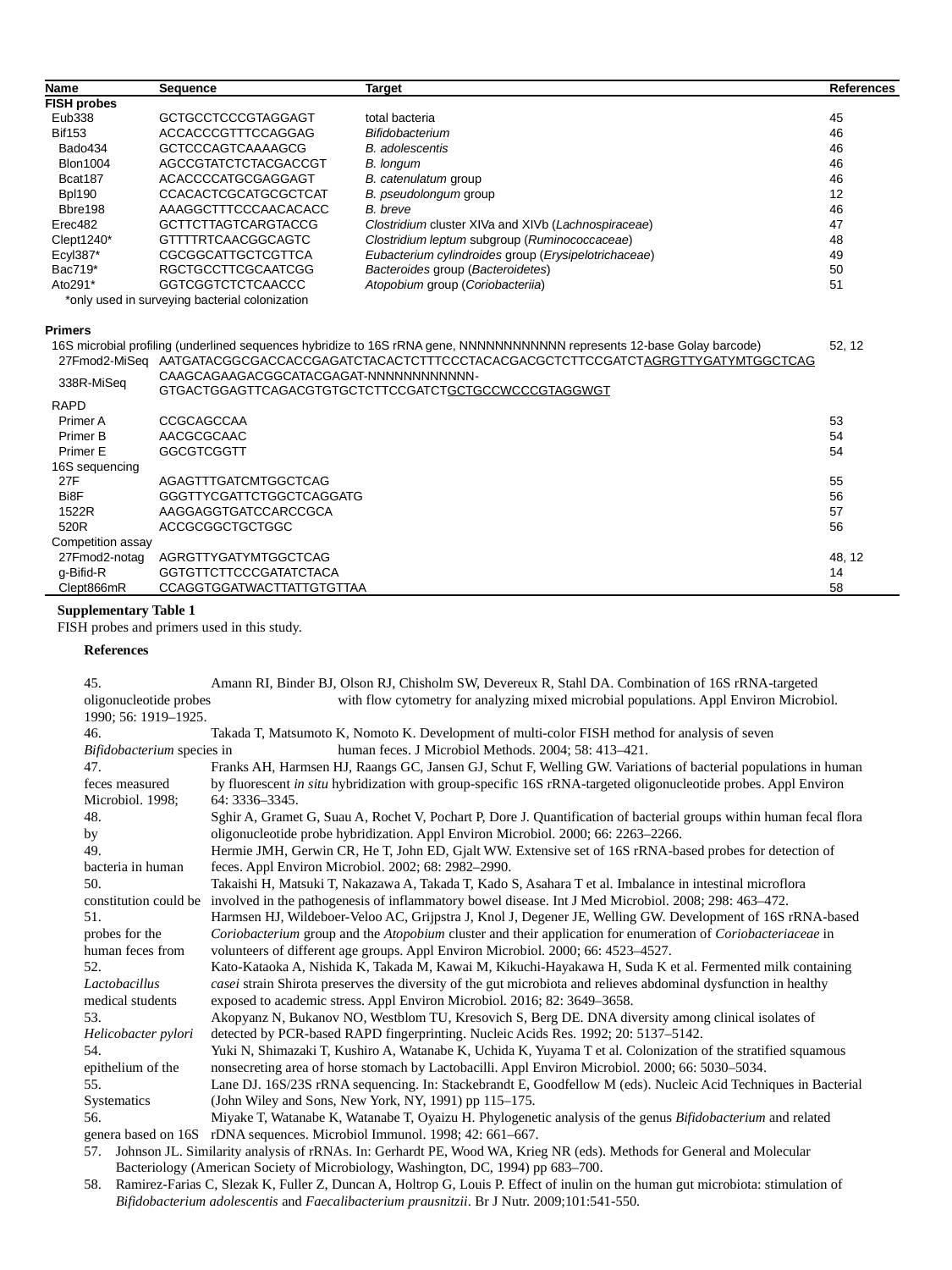

| Name | Sequence | Target | | References |
| --- | --- | --- | --- | --- |
| FISH probes | | | | |
| Eub338 | GCTGCCTCCCGTAGGAGT | total bacteria | | 45 |
| Bif153 | ACCACCCGTTTCCAGGAG | Bifidobacterium | | 46 |
| Bado434 | GCTCCCAGTCAAAAGCG | B. adolescentis | | 46 |
| Blon1004 | AGCCGTATCTCTACGACCGT | B. longum | | 46 |
| Bcat187 | ACACCCCATGCGAGGAGT | B. catenulatum group | | 46 |
| Bpl190 | CCACACTCGCATGCGCTCAT | B. pseudolongum group | | 12 |
| Bbre198 | AAAGGCTTTCCCAACACACC | B. breve | | 46 |
| Erec482 | GCTTCTTAGTCARGTACCG | Clostridium cluster XIVa and XIVb (Lachnospiraceae) | | 47 |
| Clept1240\* | GTTTTRTCAACGGCAGTC | Clostridium leptum subgroup (Ruminococcaceae) | | 48 |
| Ecyl387\* | CGCGGCATTGCTCGTTCA | Eubacterium cylindroides group (Erysipelotrichaceae) | | 49 |
| Bac719\* | RGCTGCCTTCGCAATCGG | Bacteroides group (Bacteroidetes) | | 50 |
| Ato291\* | GGTCGGTCTCTCAACCC | Atopobium group (Coriobacteriia) | | 51 |
| \*only used in surveying bacterial colonization | | | | |
| | | | | |
| Primers | | | | |
| 16S microbial profiling (underlined sequences hybridize to 16S rRNA gene, NNNNNNNNNNNN represents 12-base Golay barcode) | | | | 52, 12 |
| 27Fmod2-MiSeq | AATGATACGGCGACCACCGAGATCTACACTCTTTCCCTACACGACGCTCTTCCGATCTAGRGTTYGATYMTGGCTCAG | | | |
| 338R-MiSeq | CAAGCAGAAGACGGCATACGAGAT-NNNNNNNNNNNN-GTGACTGGAGTTCAGACGTGTGCTCTTCCGATCTGCTGCCWCCCGTAGGWGT | | | |
| RAPD | | | | |
| Primer A | CCGCAGCCAA | | | 53 |
| Primer B | AACGCGCAAC | | | 54 |
| Primer E | GGCGTCGGTT | | | 54 |
| 16S sequencing | | | | |
| 27F | AGAGTTTGATCMTGGCTCAG | | | 55 |
| Bi8F | GGGTTYCGATTCTGGCTCAGGATG | | | 56 |
| 1522R | AAGGAGGTGATCCARCCGCA | | | 57 |
| 520R | ACCGCGGCTGCTGGC | | | 56 |
| Competition assay | | | | |
| 27Fmod2-notag | AGRGTTYGATYMTGGCTCAG | | | 48, 12 |
| g-Bifid-R | GGTGTTCTTCCCGATATCTACA | | | 14 |
| Clept866mR | CCAGGTGGATWACTTATTGTGTTAA | | | 58 |
Supplementary Table 1
FISH probes and primers used in this study.
References
45.	Amann RI, Binder BJ, Olson RJ, Chisholm SW, Devereux R, Stahl DA. Combination of 16S rRNA-targeted oligonucleotide probes 	with flow cytometry for analyzing mixed microbial populations. Appl Environ Microbiol. 1990; 56: 1919–1925.
46.	Takada T, Matsumoto K, Nomoto K. Development of multi-color FISH method for analysis of seven Bifidobacterium species in 	human feces. J Microbiol Methods. 2004; 58: 413–421.
47.	Franks AH, Harmsen HJ, Raangs GC, Jansen GJ, Schut F, Welling GW. Variations of bacterial populations in human feces measured 	by fluorescent in situ hybridization with group-specific 16S rRNA-targeted oligonucleotide probes. Appl Environ Microbiol. 1998; 	64: 3336–3345.
48.	Sghir A, Gramet G, Suau A, Rochet V, Pochart P, Dore J. Quantification of bacterial groups within human fecal flora by 	oligonucleotide probe hybridization. Appl Environ Microbiol. 2000; 66: 2263–2266.
49.	Hermie JMH, Gerwin CR, He T, John ED, Gjalt WW. Extensive set of 16S rRNA-based probes for detection of bacteria in human 	feces. Appl Environ Microbiol. 2002; 68: 2982–2990.
50.	Takaishi H, Matsuki T, Nakazawa A, Takada T, Kado S, Asahara T et al. Imbalance in intestinal microflora constitution could be 	involved in the pathogenesis of inflammatory bowel disease. Int J Med Microbiol. 2008; 298: 463–472.
51.	Harmsen HJ, Wildeboer-Veloo AC, Grijpstra J, Knol J, Degener JE, Welling GW. Development of 16S rRNA-based probes for the 	Coriobacterium group and the Atopobium cluster and their application for enumeration of Coriobacteriaceae in human feces from 	volunteers of different age groups. Appl Environ Microbiol. 2000; 66: 4523–4527.
52.	Kato-Kataoka A, Nishida K, Takada M, Kawai M, Kikuchi-Hayakawa H, Suda K et al. Fermented milk containing Lactobacillus 	casei strain Shirota preserves the diversity of the gut microbiota and relieves abdominal dysfunction in healthy medical students 	exposed to academic stress. Appl Environ Microbiol. 2016; 82: 3649–3658.
53.	Akopyanz N, Bukanov NO, Westblom TU, Kresovich S, Berg DE. DNA diversity among clinical isolates of Helicobacter pylori 	detected by PCR-based RAPD fingerprinting. Nucleic Acids Res. 1992; 20: 5137–5142.
54.	Yuki N, Shimazaki T, Kushiro A, Watanabe K, Uchida K, Yuyama T et al. Colonization of the stratified squamous epithelium of the 	nonsecreting area of horse stomach by Lactobacilli. Appl Environ Microbiol. 2000; 66: 5030–5034.
55.	Lane DJ. 16S/23S rRNA sequencing. In: Stackebrandt E, Goodfellow M (eds). Nucleic Acid Techniques in Bacterial Systematics 	(John Wiley and Sons, New York, NY, 1991) pp 115–175.
56.	Miyake T, Watanabe K, Watanabe T, Oyaizu H. Phylogenetic analysis of the genus Bifidobacterium and related genera based on 16S 	rDNA sequences. Microbiol Immunol. 1998; 42: 661–667.
Johnson JL. Similarity analysis of rRNAs. In: Gerhardt PE, Wood WA, Krieg NR (eds). Methods for General and Molecular Bacteriology (American Society of Microbiology, Washington, DC, 1994) pp 683–700.
Ramirez-Farias C, Slezak K, Fuller Z, Duncan A, Holtrop G, Louis P. Effect of inulin on the human gut microbiota: stimulation of Bifidobacterium adolescentis and Faecalibacterium prausnitzii. Br J Nutr. 2009;101:541-550.

## Slide 2
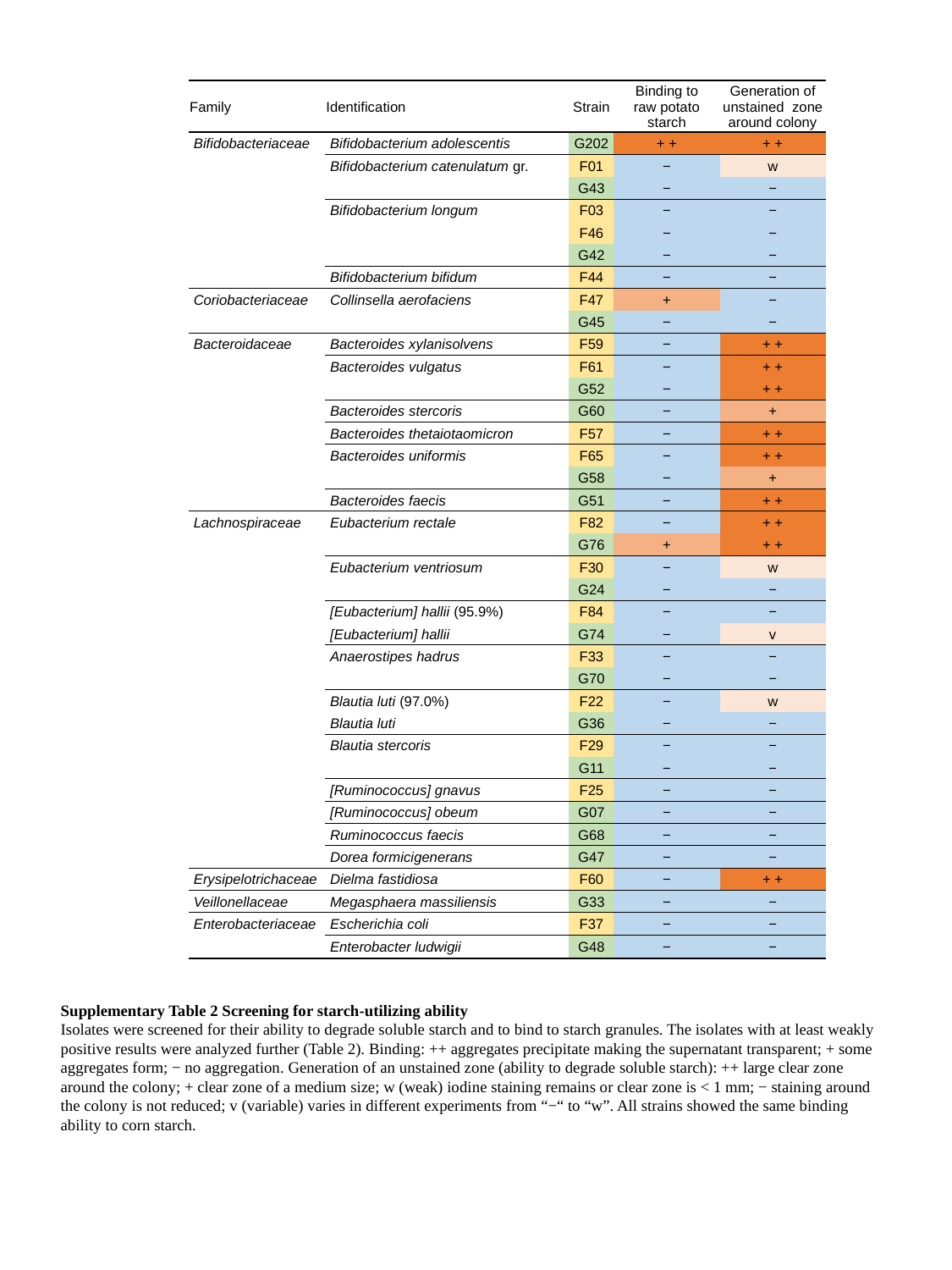

| Family | Identification | Strain | Binding to raw potato starch | Generation of unstained zone around colony |
| --- | --- | --- | --- | --- |
| Bifidobacteriaceae | Bifidobacterium adolescentis | G202 | + + | + + |
| | Bifidobacterium catenulatum gr. | F01 | − | w |
| | | G43 | − | − |
| | Bifidobacterium longum | F03 | − | − |
| | | F46 | − | − |
| | | G42 | − | − |
| | Bifidobacterium bifidum | F44 | − | − |
| Coriobacteriaceae | Collinsella aerofaciens | F47 | + | − |
| | | G45 | − | − |
| Bacteroidaceae | Bacteroides xylanisolvens | F59 | − | + + |
| | Bacteroides vulgatus | F61 | − | + + |
| | | G52 | − | + + |
| | Bacteroides stercoris | G60 | − | + |
| | Bacteroides thetaiotaomicron | F57 | − | + + |
| | Bacteroides uniformis | F65 | − | + + |
| | | G58 | − | + |
| | Bacteroides faecis | G51 | − | + + |
| Lachnospiraceae | Eubacterium rectale | F82 | − | + + |
| | | G76 | + | + + |
| | Eubacterium ventriosum | F30 | − | w |
| | | G24 | − | − |
| | [Eubacterium] hallii (95.9%) | F84 | − | − |
| | [Eubacterium] hallii | G74 | − | v |
| | Anaerostipes hadrus | F33 | − | − |
| | | G70 | − | − |
| | Blautia luti (97.0%) | F22 | − | w |
| | Blautia luti | G36 | − | − |
| | Blautia stercoris | F29 | − | − |
| | | G11 | − | − |
| | [Ruminococcus] gnavus | F25 | − | − |
| | [Ruminococcus] obeum | G07 | − | − |
| | Ruminococcus faecis | G68 | − | − |
| | Dorea formicigenerans | G47 | − | − |
| Erysipelotrichaceae | Dielma fastidiosa | F60 | − | + + |
| Veillonellaceae | Megasphaera massiliensis | G33 | − | − |
| Enterobacteriaceae | Escherichia coli | F37 | − | − |
| | Enterobacter ludwigii | G48 | − | − |
Supplementary Table 2 Screening for starch-utilizing ability
Isolates were screened for their ability to degrade soluble starch and to bind to starch granules. The isolates with at least weakly positive results were analyzed further (Table 2). Binding: ++ aggregates precipitate making the supernatant transparent; + some aggregates form; − no aggregation. Generation of an unstained zone (ability to degrade soluble starch): ++ large clear zone around the colony; + clear zone of a medium size; w (weak) iodine staining remains or clear zone is < 1 mm; − staining around the colony is not reduced; v (variable) varies in different experiments from “−“ to “w”. All strains showed the same binding ability to corn starch.

## Slide 3
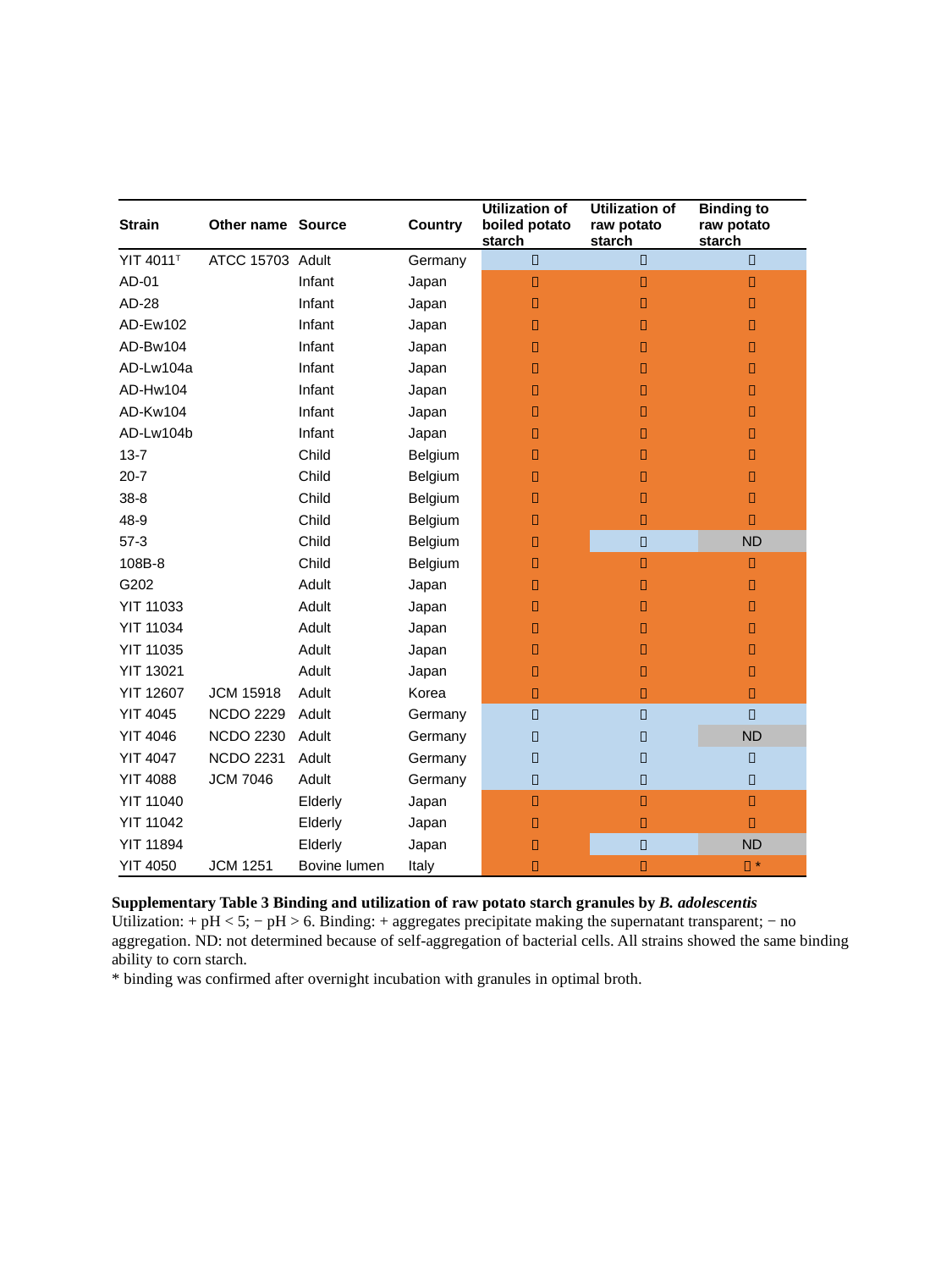

| Strain | Other name | Source | Country | Utilization of boiled potato starch | Utilization of raw potato starch | Binding to raw potato starch |
| --- | --- | --- | --- | --- | --- | --- |
| YIT 4011T | ATCC 15703 | Adult | Germany | － | － | － |
| AD-01 | | Infant | Japan | ＋ | ＋ | ＋ |
| AD-28 | | Infant | Japan | ＋ | ＋ | ＋ |
| AD-Ew102 | | Infant | Japan | ＋ | ＋ | ＋ |
| AD-Bw104 | | Infant | Japan | ＋ | ＋ | ＋ |
| AD-Lw104a | | Infant | Japan | ＋ | ＋ | ＋ |
| AD-Hw104 | | Infant | Japan | ＋ | ＋ | ＋ |
| AD-Kw104 | | Infant | Japan | ＋ | ＋ | ＋ |
| AD-Lw104b | | Infant | Japan | ＋ | ＋ | ＋ |
| 13-7 | | Child | Belgium | ＋ | ＋ | ＋ |
| 20-7 | | Child | Belgium | ＋ | ＋ | ＋ |
| 38-8 | | Child | Belgium | ＋ | ＋ | ＋ |
| 48-9 | | Child | Belgium | ＋ | ＋ | ＋ |
| 57-3 | | Child | Belgium | ＋ | － | ND |
| 108B-8 | | Child | Belgium | ＋ | ＋ | ＋ |
| G202 | | Adult | Japan | ＋ | ＋ | ＋ |
| YIT 11033 | | Adult | Japan | ＋ | ＋ | ＋ |
| YIT 11034 | | Adult | Japan | ＋ | ＋ | ＋ |
| YIT 11035 | | Adult | Japan | ＋ | ＋ | ＋ |
| YIT 13021 | | Adult | Japan | ＋ | ＋ | ＋ |
| YIT 12607 | JCM 15918 | Adult | Korea | ＋ | ＋ | ＋ |
| YIT 4045 | NCDO 2229 | Adult | Germany | － | － | － |
| YIT 4046 | NCDO 2230 | Adult | Germany | － | － | ND |
| YIT 4047 | NCDO 2231 | Adult | Germany | － | － | － |
| YIT 4088 | JCM 7046 | Adult | Germany | － | － | － |
| YIT 11040 | | Elderly | Japan | ＋ | ＋ | ＋ |
| YIT 11042 | | Elderly | Japan | ＋ | ＋ | ＋ |
| YIT 11894 | | Elderly | Japan | ＋ | － | ND |
| YIT 4050 | JCM 1251 | Bovine lumen | Italy | ＋ | ＋ | ＋\* |
Supplementary Table 3 Binding and utilization of raw potato starch granules by B. adolescentis
Utilization: + pH < 5; − pH > 6. Binding: + aggregates precipitate making the supernatant transparent; − no aggregation. ND: not determined because of self-aggregation of bacterial cells. All strains showed the same binding ability to corn starch.
* binding was confirmed after overnight incubation with granules in optimal broth.
